# A first generation whole genome RH map of the river buffalo with comparison to domestic cattle

**DOI:** 10.1186/1471-2164-9-631

**Published:** 2008-12-24

**Authors:** M Elisabete J Amaral, Jason R Grant, Penny K Riggs, Nedenia B Stafuzza, Edson A Rodrigues Filho, Tom Goldammer, Rosemarie Weikard, Ronald M Brunner, Kelli J Kochan, Anthony J Greco, Jooha Jeong, Zhipeng Cai, Guohui Lin, Aparna Prasad, Satish Kumar, G Pardha Saradhi, Boby Mathew, M Aravind Kumar, Melissa N Miziara, Paola Mariani, Alexandre R Caetano, Stephan R Galvão, Madhu S Tantia, Ramesh K Vijh, Bina Mishra, ST Bharani Kumar, Vanderlei A Pelai, Andre M Santana, Larissa C Fornitano, Brittany C Jones, Humberto Tonhati, Stephen Moore, Paul Stothard, James E Womack

**Affiliations:** 1 Department of Biologia, UNESP – São Paulo State University, IBILCE, São Jose Rio Preto, SP, Brazil; 2 Department of Agricultural, Food and Nutritional Science, University of Alberta, Edmonton, AB, Canada; 3 Department of Animal Science, Texas A&M University, College Station, TX, USA; 4 Forschungsbereich Molekularbiologie, Forschungsinstitut für die Biologie landwirtschaftlicher Nutztiere (FBN), Dummerstorf, Germany; 5 Departament of Computing Science, University of Alberta, Edmonton, AB, Canada; 6 Centre for Cellular and Molecular Biology, Hyderabad, India; 7 Parco Tecnologico Padano, Via Einstein, Polo Universitario, Lodi, Italy; 8 Embrapa Recursos Geneticos e Biotecnologia, Parque Estacao Biologica, Brasilia, DF, Brazil; 9 Programa de Pos Graduacao em Ciencias Animais, Universidade de Brasilia, Brasilia, DF, Brazil; 10 National Bureau of Animal Genetic Resources, Karnal, India; 11 Department of Veterinary Pathobiology, Texas A&M University, College Station, TX, USA; 12 Department of Animal Sciences, UNESP – São Paulo State University, FCAV, Jaboticabal, SP, Brazil

## Abstract

**Background:**

The recently constructed river buffalo whole-genome radiation hybrid panel (BBURH_5000_) has already been used to generate preliminary radiation hybrid (RH) maps for several chromosomes, and buffalo-bovine comparative chromosome maps have been constructed. Here, we present the first-generation whole genome RH map (WG-RH) of the river buffalo generated from cattle-derived markers. The RH maps aligned to bovine genome sequence assembly Btau_4.0, providing valuable comparative mapping information for both species.

**Results:**

A total of 3990 markers were typed on the BBURH_5000 _panel, of which 3072 were cattle derived SNPs. The remaining 918 were classified as cattle sequence tagged site (STS), including coding genes, ESTs, and microsatellites. Average retention frequency per chromosome was 27.3% calculated with 3093 scorable markers distributed in 43 linkage groups covering all autosomes (24) and the X chromosomes at a LOD ≥ 8. The estimated total length of the WG-RH map is 36,933 cR_5000_. Fewer than 15% of the markers (472) could not be placed within any linkage group at a LOD score ≥ 8. Linkage group order for each chromosome was determined by incorporation of markers previously assigned by FISH and by alignment with the bovine genome sequence assembly (Btau_4.0).

**Conclusion:**

We obtained radiation hybrid chromosome maps for the entire river buffalo genome based on cattle-derived markers. The alignments of our RH maps to the current bovine genome sequence assembly (Btau_4.0) indicate regions of possible rearrangements between the chromosomes of both species. The river buffalo represents an important agricultural species whose genetic improvement has lagged behind other species due to limited prior genomic characterization. We present the first-generation RH map which provides a more extensive resource for positional candidate cloning of genes associated with complex traits and also for large-scale physical mapping of the river buffalo genome.

## Background

Among domestic animals, the water buffalo (*Bubalus bubalis*), particularly the river buffalo, holds great promise and potential for animal production. According to estimates by the "Food and Agriculture Organization of the United Nations" the global water buffalo population has increased 98% in the last decades, from 88 million in 1961 to 174 million in 2005. Buffalo is the most important farm animal species in Asia, especially India, where it is extensively used for milk, meat, fuel and fertilizer production (from manure), as well as for draught power [1]. Currently, river buffalo can be found in many countries worldwide. The growth of its population outside of the Asian continent is mainly related to the increasing interest in milk production used to produce cream, butter, yogurt and many cheeses. Brazil, for instance, is the largest buffalo breeding center outside the Asian continent holding the largest buffalo herd in the Americas.

River buffalo, along with domestic cattle, belongs to the subfamily Bovinae whereas sheep and goat belong to the subfamily Caprinae, all members of the family Bovidae. These species have been shown to be closely related, sharing homology in chromosome banding [2-5] and gene mapping [6-9], and have been cytogenetically characterized in detail.

Cattle (*Bos taurus*, BTA) and river buffalo (*Bubalus bubalis*, BBU) chromosomes can be matched arm for arm at the cytogenetic level [9-12]. While the cattle genome consists of 29 acrocentric autosomes and a pair, X/Y, of sexual chromosomes, the river buffalo genome has 5 biarmed and 19 acrocentric autosomes plus the X and Y chromosomes [13]. All buffalo chromosomes arms have homology to single bovine acrocentric chromosomes. BBU1 appears to be a fusion of BTA1 and 27, BBU2 equals BTA2 and 23, BBU3 equals BTA8 and 19, BBU4 equals BTA5 and 28, and BBU5 equals BTA16 and 29 at the cytogenetic level with state of the art banding. All the other chromosomes have a one to one correspondence between the two species [9,14].

Although the latest cytogenetic map of the river buffalo genome reports 388 FISH-mapped loci [11], much remains to be done in order to generate high resolution maps of the buffalo genome.

The radiation hybrid (RH) mapping approach has been established as the method of choice to generate medium to high resolution maps. RH panels are available for several domestic mammalian species such as cow [15], pig [16], horse [17,18], dog [19] and cat [20]. The production of a RH panel in river buffalo is quite recent [21]. It has been used to construct preliminary RH maps for individual buffalo chromosomes, BBU1 [22]; BBU3 and 10 [21]; BBU7 [23]; BBU6 [24] and BBUX [25]. These preliminary maps, based on cattle-derived markers, demonstrated that the bovine genome is a useful source of markers for the buffalo genome mapping allowing rapid and efficient transfer of information from cattle to buffalo.

Taking advantage of the extensive resources and tools now available as a result of the bovine genome sequencing project, and given the close evolutionary relationship between cattle and river buffalo, the opportunity was available for study of the buffalo genome on a large scale to detect micro rearrangements in the marker order that might have taken place during the evolutionary divergence of these species. Of particular importance is the comparison of gene order between the two ruminant species, buffalo and cattle, and an assessment of rearrangements which is independent of previous, more limited comparisons done with specific markers of the two species by somatic hybrid cell analysis and FISH.

A high resolution genome map of buffalo will be an important tool for evaluating chromosomal evolution among species of Bovidae which, according to several phylogenetic studies, are separated by only a few million years [26-28]. It will therefore facilitate extrapolation of data from cattle genomics and at some point aid in the development of additional genomic tools for buffalo. Here, we report the use of the BBURH_5000 _panel to construct the first-generation whole genome radiation hybrid map (WG-RH) of the river buffalo containing more than 2500 cattle-derived loci covering all autosomes and the X chromosome.

## Results and discussion

In this report, we present the first-generation whole genome radiation hybrid map of the river buffalo (BBU WG-RH). From the total of 3093 markers used to assemble the maps, 472 could not be placed on the maps, so 2621 are included on the RH maps of 24 autosomes and the X chromosome.

This is the first genome-wide RH map of the river buffalo and establishes a base genomic map from which higher resolution maps can be generated in the future. This first generation map also provides a characterization of the BBURH_5000 _panel allowing estimation of its potential limit of resolution. The BBU WG-RH map spans a total of 36,933 cR. However, accurate physical distances are not available for the genome lengths spanned by this WG-RH map. Because karyotype analysis indicates extensive similarity at the level of chromosome arms between buffalo and cattle, and presuming a genome size of 3000 Mbp in both species, we extrapolate the physical distance of the buffalo genome to be approximately 2623 Mbp, providing an estimate of 73.6 kb/cR_5000_. The BBURH_5000 _panel clones retained buffalo DNA with an average retention rate of 27.3% and 73.6 kb/cR_5000_. A summary of the river buffalo WG-RH map statistics is shown in Additional file 1.

Markers were distributed into linkage groups based on two-point LOD score threshold of ≥ 8 to assign linkage to a particular group of markers and not to others located on different chromosomes. A total of 43 linkage groups were generated for the whole genome. The number of markers mapped to each chromosome varied from 49 (BBU22) to 233 (BBU2). The RH maps from the bi-armed chromosomes presented at least two linkage groups (one for each arm) with the exception of BBU 5, in which markers were distributed into a single linkage group. Among the acrocentric chromosomes, the number of linkage groups ranged from 1–5 with BBU8 containing the greatest number of linkage groups (5), but also had the fewest mapped cattle-derived STS markers (2). Considering that the markers mapped on BBU8 are almost entirely derived from cattle SNPs, the increased number of linkage groups observed might be related to the irregular distribution of the SNPs on the bovine chromosome 4, which is known to be homologous to BBU8. The number of linkage groups and the total number of markers per chromosome is also presented in Additional file 1.

**Figure 1:**
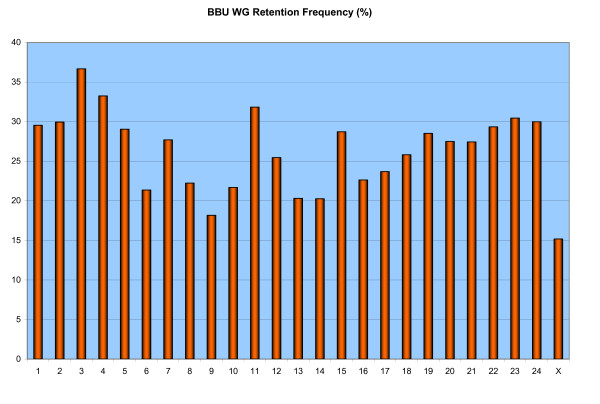
**BBU WG retention frequency per chromosome**. Marker retention frequency across the entire river buffalo genome.

The average retention frequency (RF) for the data set is 27.3%, with the frequency for individual autosomes varying from 18.9% on BBU9 to 37.2% on BBU3, which contains the selectable marker thymidine kinase (Additional file 1). The average retention frequency per chromosome observed on the BBURH panel is similar to the estimates reported for other 5000 rad panels such as those constructed for cattle [15], horse [29] and dog [30]. The relatively low RF for BBUX (15.6%) was expected, since the buffalo parental cell line was created from a male animal. The variation in retention frequency for each chromosome is shown graphically in figure 1.

**Figure 2:**
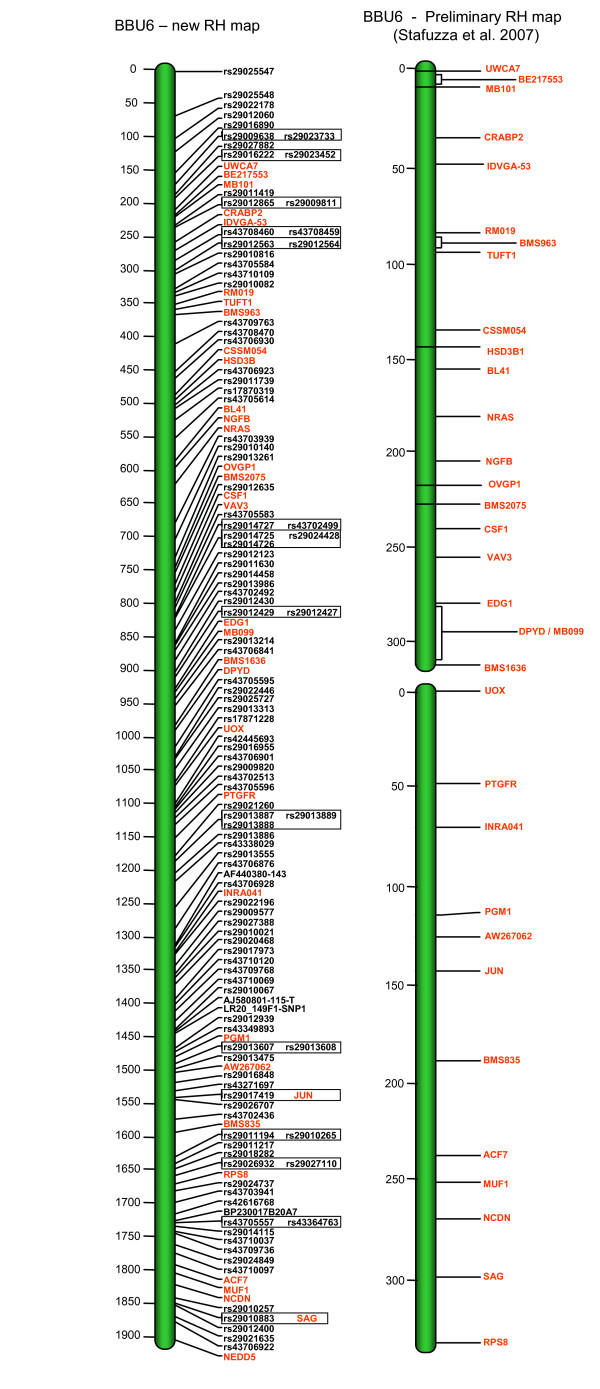
**Example of the additional number of markers incorporated on BBU6 RH map**. This figure shows a comparison between the preliminary and the current BBU6 RH map. New markers incorporated in the first-generation RH map are in black.

Preliminary RH maps for individual BBU chromosomes (BBU1, 3, 6, 7, 10 and X) have been previously published [21-25]. The new maps presented herein demonstrate a considerable increase in the number of mapped markers resulting in maps with greatly improved coverage over the previously published data. In addition to 19 chromosome maps published for the first time, the remaining six chromosomes are presented with the following number of additional markers: BBU1 (+133), BBU3 (+141), BBU6 (+114), BBU7 (+ 84), BBU10 (+74) and BBUX (+ 31). An example is shown in figure 2 with BBU6 illustrating the improvement regarding the marker density obtained with the new RH map. In general, the order of the markers between the new and the previous RH maps shows a high level of agreement except for minor flips involving closely linked loci.

To date, this is the first whole genome RH and comparative map produced for the river buffalo. Also, this is the first report illustrating the extensive use of cross-species oligo assays to produce RH maps, a technique which contributed significantly to the total number of markers placed on the maps. In addition, this BBU WG-RH map, containing 2621 markers, is the most dense among the first-generation RH maps produced for other domestic species, such as pig (757 markers) [31], cattle (1087 markers) [32], horse (730 markers) [29], cat (600 markers) [33] and dog (400 markers) [30]. In addition, this map was assembled with fewer RH linkage groups (43) than previously-reported for first-generation RH maps in cattle (61) [32], pig (128) [31] and horse (101) [29].

**Figure 3:**
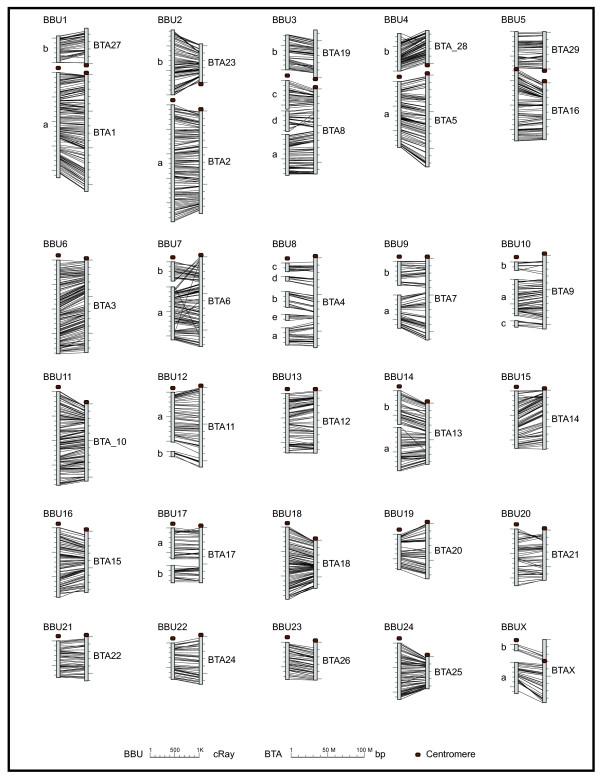
**Overview of the WG-RH map in comparison with their respective homologous chromosome in BTA**. For each comparison the buffalo chromosome is on the left and the homologous bovine chromosome is on the right. If a buffalo chromosome is represented by more then one linkage group, they are labeled alphabetically, with 'a' being the largest linkage group. Lines between the maps connect markers common in both maps. Distances on the buffalo chromosomes are scaled in cR and always start at the top of the map except for linkage group 'b' in maps BBU1-4, which start at the bottom. Distances on the bovine chromosome are scaled in bp and always start at the centromere.

Because traditional genetic maps are not currently available for river buffalo, we compared the order of the markers from the BBU-WG RH maps to the current bovine genome sequence assembly (NCBI Btau_4.0). Figure 3 shows a schematic overview of the WG-RH map in comparison with their respective homologous chromosome in BTA. To be able to extrapolate the information between the genomes it is necessary to align conserved segments accurately between species. Mapping a large number of markers on the buffalo genome and cross-referencing these with the map locations for the markers in the bovine achieved this alignment.

**Figure 4:**
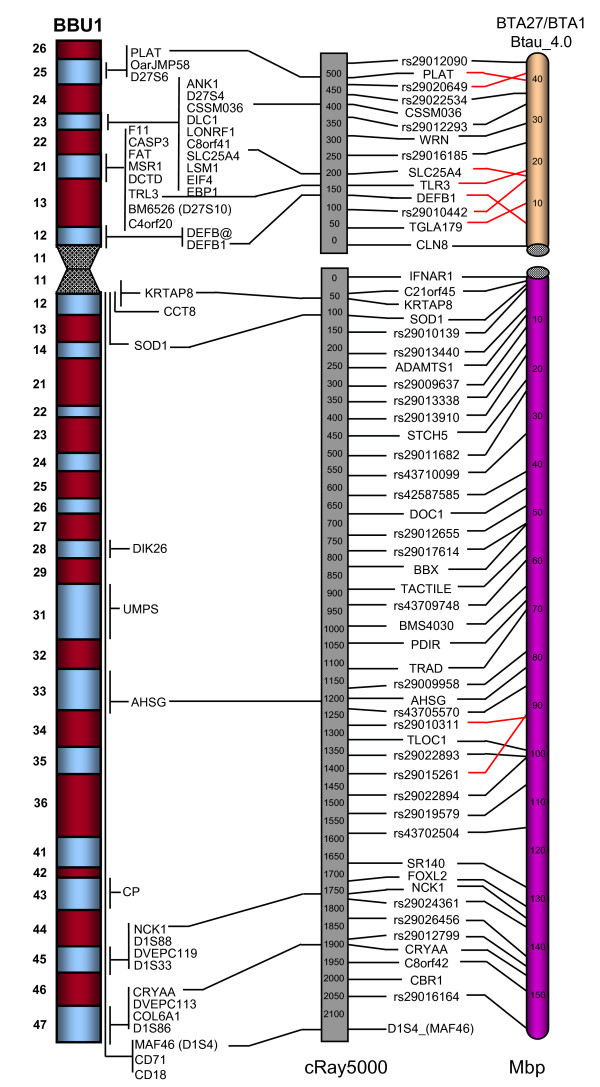
**Comparison of the BBU1 RH map, the latest cytogenetic map and the alignment with the BTA1 and BTA27 sequence assembly (Btau_4.0)**. The BBU1 RH map is shown in the centre, the G-banded ideogram on the left and the corresponding cattle chromosomes on the right. The distances in cR5000 and Mbp are shown below each corresponding map. Considering the large number of mapped markers, only one marker per 50 cR is shown in the RH map. The BTA1 and BTA27 sequence maps shows one marker every 10 Mbp. Markers common to both BBU RH and the cattle sequence are joined by a solid black line or a solid red line. Solid red lines indicate markers which are oriented sequentially regarding the cattle but inverted. A solid black line also joins those markers on the BBU RH map that have been physically mapped by FISH to their location on the ideogram (Di Meo et al. 2008).

Sixty-eight markers previously assigned by fluorescence *in situ *hybridization (FISH) [11] were incorporated into the RH maps serving as anchor markers for the RH maps to correctly orient the linkage groups. All chromosomes, except BBU15, had at least one marker previously mapped by FISH represented on the respective RH map. Figure 4 shows in detail the comparative mapping between the BBU1 RH map, the latest G-banded ideogram of the river buffalo chromosome [11] and the alignment with the bovine genome sequence assembly (Btau_4.0). Considering the large number of mapped markers, only one marker per 50 cR is presented in the RH map figure for better illustration. The individual BBU RH comparative maps from the entire buffalo genome are displayed in additional file 2. Also, a complete version of the RH maps containing all the mapped markers can be viewed at the National Center for Biological (NCBI) river buffalo genome page .

The comparison revealed few disagreements between the BBU RH maps, the cytogenetic map and the bovine sequence assembly. Marker order within the linkage groups for the buffalo chromosomes was consistent with the bovine genome assembly and, where information was available, in agreement with the cytogenetic assignment. As indicated on Figure 4, few discrepancies on the markers order were observed. Interestingly, most of the disagreements observed with the bovine sequence assembly, for example, those observed on BBU4, BBU5 and BBU12, involved markers derived from cattle SNPs. An exception was observed on the BBU7 RH map, which showed disagreements on the position of five genes, *KLHL8*, *TRAM1L1 *and *UGDH *from LG7a and, *GPR103 *and *TKL2 *from LG7b. These discrepancies in marker order within conserved segments might indicate small chromosome rearrangements, but could also be due to the insufficient resolution of the RH map in specific regions of the chromosomes or mistakes in the bovine sequence assembly. The number of observed disagreements in marker order positions among our RH maps and the bovine sequence and the river buffalo cytogenetic assignment may contribute to improved maps for buffalo as well as maps for other members of the Bovidae family.

Considering the limited genomic resources available for the river buffalo, the comparative mapping information presented here can be used to identify chromosomal regions potentially associate with traits that have been genetically mapped in other livestock. With the availability of the bovine genome sequence assembly it was possible to align the buffalo RH maps with the bovine genome and to obtain a large amount of information on the markers likely to also be found at a particular chromosomal location in buffalo.

## Conclusion

We have built the first-generation radiation hybrid map of the river buffalo (*Bubalus bubalis*) genome using the BBURH_5000 _panel and cattle-derived markers. Considering that genetic maps are absent for river buffalo, our goal was to provide a resource for positional candidate cloning of genes associate with complex traits and also for large-scale physical mapping of the river buffalo genome. The comparison with the bovine sequence assembly provides information sufficient for genome-wide scans to detect chromosomal regions contributing to economically important traits in river buffalo.

## Methods

### Selection of the markers

In order to link the BBU WG-RH map to the bovine genome sequence, only cattle-derived markers were used. Markers were selected from published cattle linkage and RH maps based on their location on cattle chromosomes homologous to buffalo chromosomes. A total of 3990 markers were typed on the BBURH_5000 _panel, of which 3072 were cattle-derived SNP (Single Nucleotide Polymorphism), originated from Oligo pooled assays (OPA) synthesized and assembled by Illumina Inc. (San Diego, CA). These SNPs were a subset of previously characterized bovine SNPs [34] and were selected to be evenly distributed along the bovine genome (Btau_2.0).

The remaining 918 markers, classified as cattle "sequence tagged site" (STS), included bovine coding genes, ESTs (expressed sequence tags) and microsatellites. All markers used and their information details are available at the National Center for Biological (NCBI) Information database (ProbeDB)  Additional file 3 contains the identification numbers of the markers displayed on the database.

### RH Vectors

RH vectors were produced by one of three different detection methods: conventional gel-based scoring, PCR dissociation curve analysis, or Illumina SNP analysis (detailed below).

The Illumina SNP-based RH vectors were generated by computer calling and by manual calling. RH vectors of markers derived from cattle STS were mostly generated using agarose gel analysis, except markers from BBU2 and BBU20, which were genotyped using dissociation curve analysis.

### RH panel genotyping based on agarose gels

DNA obtained from each RH cell line was diluted to a concentration of 25 ng/ul. The markers were typed on DNA from the 90 radiation hybrid lines together with control bovine and hamster DNA by PCR in 96-well microtiter plates. Each PCR reaction was performed in 10-μl reaction mixtures containing 50 ng of DNA; 1.5 mM MgCl_2_; 10 mM Tris-HCl; 50 mM KCl; 0.2 mM dGTP, dTTP, dATP and dCTP; 10 pmol each forward and reverse primer and 0.5 U of Taq DNA polymerase (AmpliTaq Gold; PE Applied Biosystems, Foster City, CA, USA). The reactions were performed in 96-well PCR plates on thermal cyclers with thermal gradient software, where available. PCR conditions included 95°C for 10 min; 35 cycles of 95°C for 30 sec, 65°C for 30 sec and 72°C for 30 sec; with a final extension cycle of 72°C for 7 min. PCR products were visualized on 2% agarose gels in 1.0× TBE buffer and stained with ethidium bromide. Each primer was typed twice on the RH panel to insure reproducibility. Strong amplification products were scored as (1), weak products as (2), and absence of amplification products was assigned as (0). Markers with discrepancies between the results from the first two runs were retyped a third time. Scores from each RH clone for each primer was entered into a Microsoft Excel spread sheet.

### RH panel genotyping based on Dissociation Curve Analysis

Real-time PCR was performed in a 20 μl reaction containing 20 ng template DNA, 1X PowerSYBR^® ^Green PCR master mix (Applied Biosystems, Foster City, CA) and 300 nM primers[35]. Amplification was carried out in 96-well plates in either a 7900HT or a 7500 sequence detection system (Applied Biosystems) with the manufacturer's default thermal profile (50°C for 2 minutes, 95°C for 10 minutes, and 40 cycles of 95°C for 15 seconds and 60°C for 1 minute) followed by a dissociation stage (95°C for 15 seconds, 60°C for 15 seconds, followed by a slow ramp to 95°C) The incubation at 50°C was not necessary, but the default profile was not changed. To test a semi-automated method, 10 μl reactions containing 10 ng template DNA were set up in 384-well format by a Precision 2000 Plus automated microplate pipetting system (Bio-Tek Instruments, Inc., Winooski, VT) and amplified in a 7900HT sequence detection system with the same thermal profile as described above. Amplification and dissociation data were analyzed with SDS software v.2.2.2 (Applied Biosystems). Radiation hybrid clones were scored independently by two people for presence or absence of the peak representing the river buffalo product. The scores were compared and discrepancies that were not clerical errors were scored as questionable.

### RH panel genotyping based on cattle SNP

DNA from the 90 cell lines of the BBURH_5000 _panel as well as hamster and bovine control DNA were typed using the Illumina BeadStation 500G genotyping system [36]. The presence or absence of SNP markers in the hybrids was determined using two methods: manual and computer scoring. Manual scoring was performed according to methods previously described [34]. Briefly, Illumina BeadStudio (Version 3) software was used to visualize all the hybrids and the controls for a single marker with a cartesian plot where the X- and Y-axis represent the intensities of the A and B allele, respectively. Hybrids form distinct clusters depending on the presence or absence of the marker and when compared to the positive and negative controls, this allows easy scoring. Although much faster then conventional scoring by PCR, this method can still take considerable time to perform for thousands of markers. We sought to automate this process through computer scoring. We created a Java program to analyze the intensities of the A and B alleles from the Illumina data and compare them to the positive and negative controls. There were 864 and 2075 markers typed by manual and computer methods, respectively. Using markers that were in common to both methods (755 markers), the accuracy of computer scoring was determined. Compared to manual calls, computational scoring was 98.0% accurate at determining present calls and 98.8% accurate for absent calls.

### Mapping of markers against the bovine sequence assembly

Genomic sequence coordinates for the SNP markers were determined by performing BLAST [37] comparisons of the SNP flanking sequences against the latest bovine genome assembly (Btau_4.0 – ) [38], using an expect threshold of 1e-50. Positions of the STS markers on Btau_4.0 were obtained by performing *in-silico *PCR  with primers designed for the STS markers.

### RH data analysis

Of the 3093 markers that generated RH vectors, 2621 were assigned to 43 linkage groups of 5 markers or more based on two-point analysis using CarthaGene [39]. A minimum LOD score of 8 was used as evidence of linkage. RH linkage groups were assigned to chromosomes based on a comparison of the markers in each group with the position of the markers on Btau_4.0. The buffalo chromosome was then determined by knowing the respective homologous chromosome in BTA [11]. Where more then one linkage group was associated with a chromosome, the groups were named by size (a, b, c, etc.), with 'a' having the most markers. The marker order of each linkage group was determined as described previously [40]. Briefly, RH maps were constructed using the comparative mapping approach of the CarthaGene software package [39,41]. This approach takes advantage of the known marker order in a closely related completely sequenced organism. The reference order used in this analysis was the order of the SNP and STS markers in the latest bovine genome assembly (Btau_4.0). Markers with compatible retention patterns (double markers) were merged together. RH maps were first generated by converting the RH data into a "Traveling Salesman Problem" and solving using the Lin-Kernighan heuristic based commands: lkh, lkhn, lkhl, and lkhd [42]. The greedy command was used on each RH linkage group which tries to improve the marker order using a taboo search algorithm. Each map was refined further by iteratively testing all marker permutations in a sliding window of size 7 (flips) and then testing the reliability of the map by displacing each marker in all possible intervals (polish). While maps were initially generated using the haploid equal retention model, all final map distances, except BBUX, were generated using the diploid equal retention model. Final maps were drawn using CMap .

## Authors' contributions

MEJA and JEW coordinated the project, made the BBURH_5000 _panel, carried out genotyping, screened the RH panel, and drafted the manuscript. JRG, PS, ZC, AP and GL built the RH maps, performed map and sequence comparisons and helped draft the manuscript. PKR produced and analyzed RH scoring and helped draft the manuscript. NBS and EARF carried out genotyping and designed the comparative mapping figures. SM, TG, RW, RMB, KJK, AJG, JJ, SK, GPS, BM, MAK, MST, VAP, MNM, LCF, AMS, BCJ, HT, ARC, SRG, and PM carried out genotyping of RH panel. All authors read and approved the final manuscript.

## Supplementary Material

Additional file 1 **BBURH_5000 _map statistics by chromosome**. A table containing the summary of the river buffalo WG-RH map statistics. Click here for file

Additional file 2 **Comparative maps from the buffalo genome (24 autosomes and the X chromosome), including the RH maps, the latest G-banded ideogram of the river buffalo chromosomes and the alignment with the bovine genome sequence assembly (Btau_4.0)**. The RH maps are shown in the centre, the G-banded ideogram on the left and the corresponding cattle chromosomes on the right. The distances in cR5000 and Mbp are shown below each corresponding map. For better illustration, the BBU RH maps of the autosomes shows one marker per 50 cR and the BTA sequence maps shows one marker every 10 Mbp. Markers common to both BBU RH and the cattle sequence are joined by a solid black line or a solid red line. Solid red lines indicate markers which are oriented sequentially regarding the cattle but inverted. A solid black line also joins those markers on the BBU RH map that have been physically mapped by FISH to their location on the ideogram (Di Meo et al. 2008). Click here for file

Additional file 3 **Identification numbers of the markers displayed on NCBI/Probe database**. In the file, first column is ProbeDB identification number, second column is submission's accession number, third column is submission's version and fourth column is markers tracking names. Click here for file
