# Supplementary material for: A first generation whole genome RH map of the river buffalo with comparison to domestic cattle
Source: BMC Genomics. 2008 Dec 24;9:631. doi: 10.1186/1471-2164-9-631 (PMC2625372; doi:10.1186/1471-2164-9-631)
Supplement: Additional file 1 — BBURH5000 map statistics by chromosome. A table containing the summary of the river buffalo WG-RH map statistics. [file 1471-2164-9-631-S1.doc]

## Table 1 - BBURH_5000_ map statistics by chromosome

| **BBU chr.** | **Length (cR)^a^** | **Phys. Length (Mbp)^b^** | **Kb/cR** | **Total No. of Markers** | **No. of Linkage Groups** | **No. of Positions** | **cR/**  **marker** | **Mbp/**  **marker** | **Retention Freq. per chr. (%)** | **Minimum Retention Freq.** | **Corresponding marker(s)** | **Maximum Retention Freq. (%)** | **Corresponding marker(s)** |
| --- | --- | --- | --- | --- | --- | --- | --- | --- | --- | --- | --- | --- | --- |
| **1** | 2694.5 | 209 | 78 | 202 | 2 | 180 | 15.0 | 1.2 | 30.1 | 16.7 | AF440368-538 | 55.6 | C21orf45 |
| **2** | 3714.1 | 194 | 52 | 233 | 2 | 215 | 17.3 | 0.9 | 30.5 | 11.1 | rs29020754,rs29023449 | 64.4 | MRPS14 |
| **3** | 2554.2 | 182 | 71 | 194 | 4 | 184 | 13.9 | 1.0 | 37.2 | 17.8 | rs41573796,rs29014468 | 86.7 | rs29021691 |
| **4** | 2113.4 | 171 | 81 | 182 | 2 | 152 | 13.9 | 1.1 | 33.9 | 18.9 | rs29013289 | 52.2 | BZ900694 |
| **5** | 2232.0 | 129 | 58 | 150 | 1 | 140 | 15.9 | 0.9 | 29.5 | 13.3 | rs29026627 | 54.4 | BZ919809 |
| **6** | 1914.8 | 127 | 66 | 147 | 1 | 131 | 14.6 | 1.0 | 21.7 | 10.0 | rs43338029 | 42.2 | NRAS |
| **7** | 1495.9 | 122 | 82 | 134 | 2 | 115 | 13.0 | 1.1 | 28.5 | 16.7 | rs29013798 | 51.1 | NUP54 |
| **8** | 1060.4 | 124 | 117 | 85 | 5 | 77 | 13.8 | 1.6 | 22.9 | 11.1 | rs29025407,rs29024797 | 45.6 | rs29011356 |
| **9** | 1170.0 | 112 | 96 | 86 | 2 | 74 | 15.8 | 1.5 | 18.9 | 12.2 | rs43527554 | 34.4 | COMP |
| **10** | 1051.9 | 108 | 103 | 85 | 3 | 79 | 13.3 | 1.4 | 22.5 | 13.3 | rs29010240 | 38.9 | ETH225_(MB009) |
| **11** | 1912.3 | 106 | 55 | 137 | 1 | 122 | 15.7 | 0.9 | 32.6 | 18.9 | btcn21374,rs29013995 | 53.3 | rs29013479 |
| **12** | 1127.1 | 110 | 98 | 72 | 2 | 61 | 18.5 | 1.8 | 26.0 | 16.7 | rs29013299 | 37.8 | rs29025220 |
| **13** | 1215.5 | 85 | 70 | 82 | 1 | 69 | 17.6 | 1.2 | 21.1 | 13.3 | rs29012179,btcn36414 | 32.2 | rs43769448 |
| **14** | 1573.8 | 84 | 53 | 94 | 2 | 78 | 20.2 | 1.1 | 21.0 | 11.1 | YWHAB | 32.2 | PLCB4 |
| **15** | 1173.4 | 81 | 69 | 73 | 1 | 64 | 18.3 | 1.3 | 29.2 | 17.8 | BMS108,rs43702464,rs29012827 | 40.0 | MAF |
| **16** | 1419.3 | 84 | 59 | 88 | 1 | 80 | 17.7 | 1.1 | 23.1 | 13.3 | rs29023696,rs29015662 | 36.7 | btcn34158 |
| **17** | 983.4 | 76 | 77 | 65 | 2 | 60 | 16.4 | 1.3 | 24.8 | 11.1 | rs29022010 | 37.8 | TLR2 |
| **18** | 1474.8 | 66 | 45 | 98 | 1 | 95 | 15.5 | 0.7 | 26.1 | 17.8 | RPL28 | 40.0 | SF3B3 |
| **19** | 723.7 | 75 | 104 | 58 | 1 | 47 | 15.4 | 1.6 | 29.1 | 17.8 | rs29013890 | 50.0 | BM3517 |
| **20** | 1158.4 | 69 | 60 | 63 | 1 | 62 | 18.7 | 1.1 | 28.0 | 17.8 | rs29012450 | 36.7 | ETH131,DIK2821,rs43706889 |
| **21** | 748.4 | 61 | 82 | 51 | 1 | 49 | 15.3 | 1.2 | 27.8 | 15.6 | rs29023702 | 44.4 | CSSM026 |
| **22** | 742.7 | 65 | 88 | 49 | 1 | 48 | 15.5 | 1.4 | 29.7 | 18.9 | rs43705590 | 41.1 | YES1 |
| **23** | 761.2 | 51 | 67 | 53 | 1 | 48 | 15.9 | 1.1 | 30.9 | 22.2 | rs42111024 | 44.4 | ACTA2 |
| **24** | 1148.4 | 44 | 38 | 76 | 1 | 76 | 15.1 | 0.6 | 30.2 | 17.8 | rs29013255 | 40.0 | RAB26,NUBP2,C160rf72 |
| **X** | 770.1 | 88 | 114 | 64 | 2 | 58 | 13.3 | 1.5 | 15.6 | 7.8 | UREB1 | 31.1 | rs29018822 |
| **Total** | **36933.7** | **2623** |  | **2621** | **43** | **2364** |  |  |  |  |  |  |  |
| **Average (Autosomes)** |  |  | **73.6** |  |  |  | **15.9** | **1.2** | **27.3** |  |  |  |  |

^a^ Values are estimated due to gaps in the map.

^b^ The size of individual chromosomes in megabase (Mbp) is deduced from their correspondence with the bovine genome (Di Meo et al. 2008) and presuming a genome size of 3000 Mbp.
