# Supplementary figures and images for: A first generation whole genome RH map of the river buffalo with comparison to domestic cattle
Source: BMC Genomics. 2008 Dec 24;9:631. doi: 10.1186/1471-2164-9-631 (PMC2625372; doi:10.1186/1471-2164-9-631)

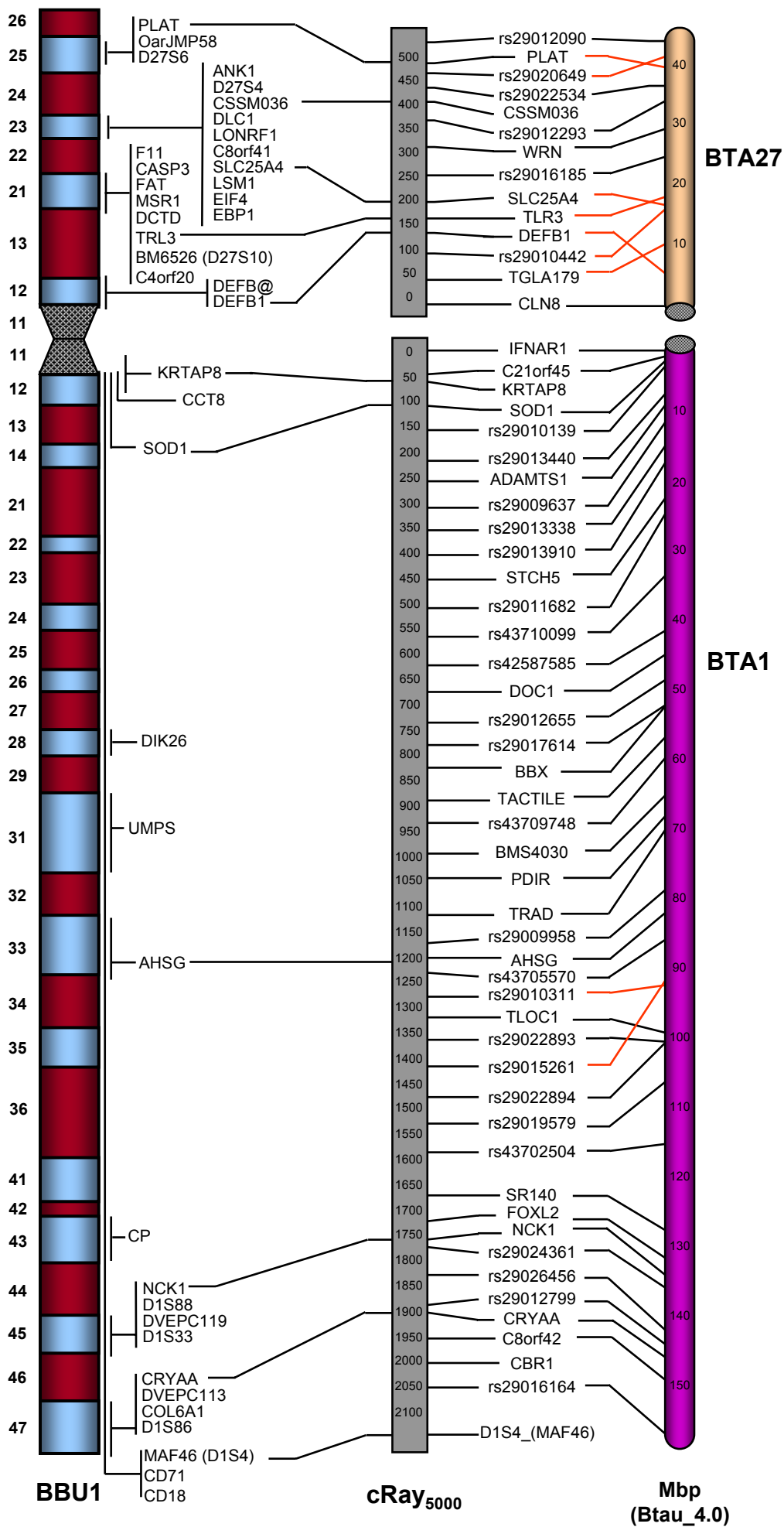

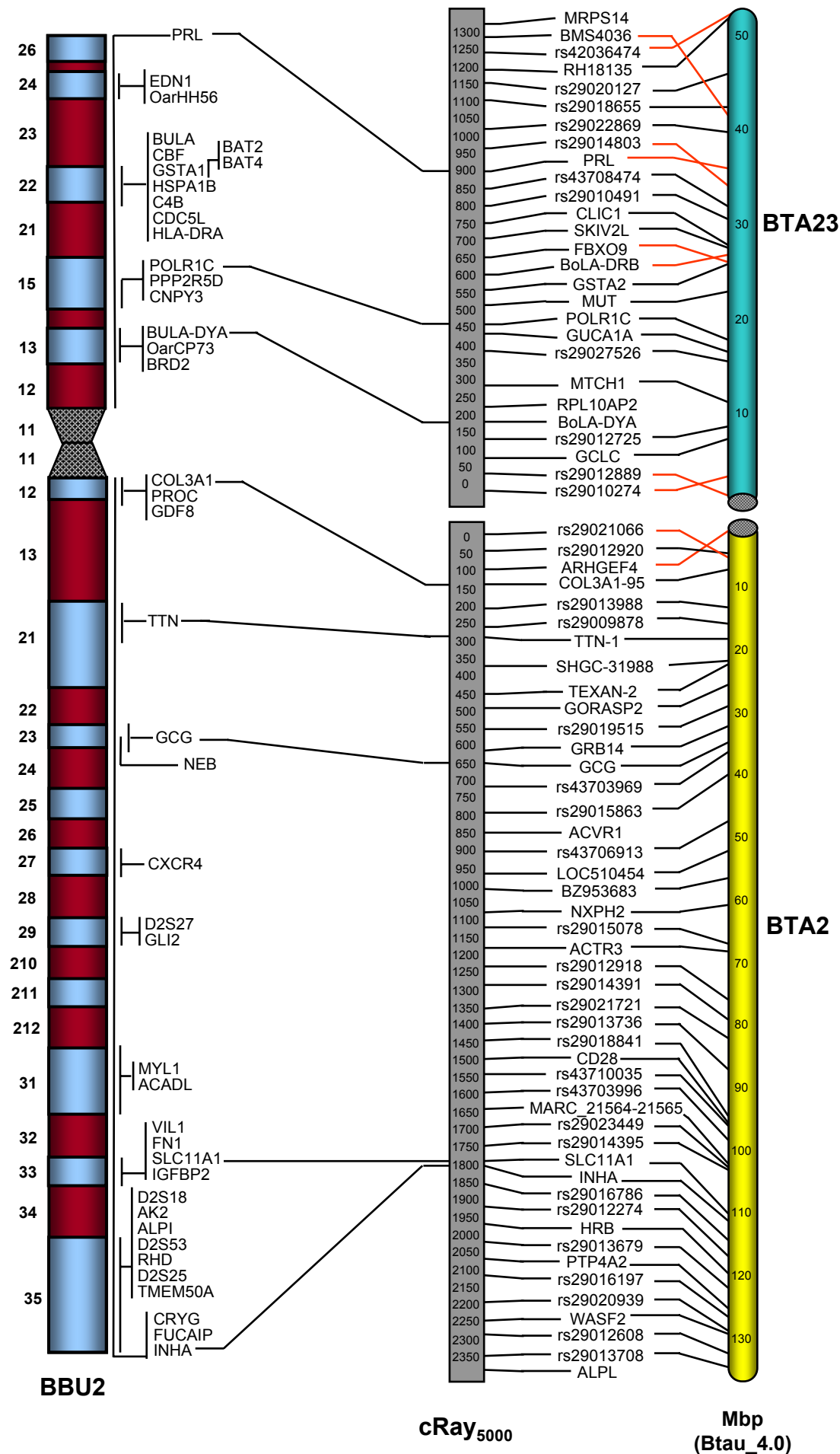

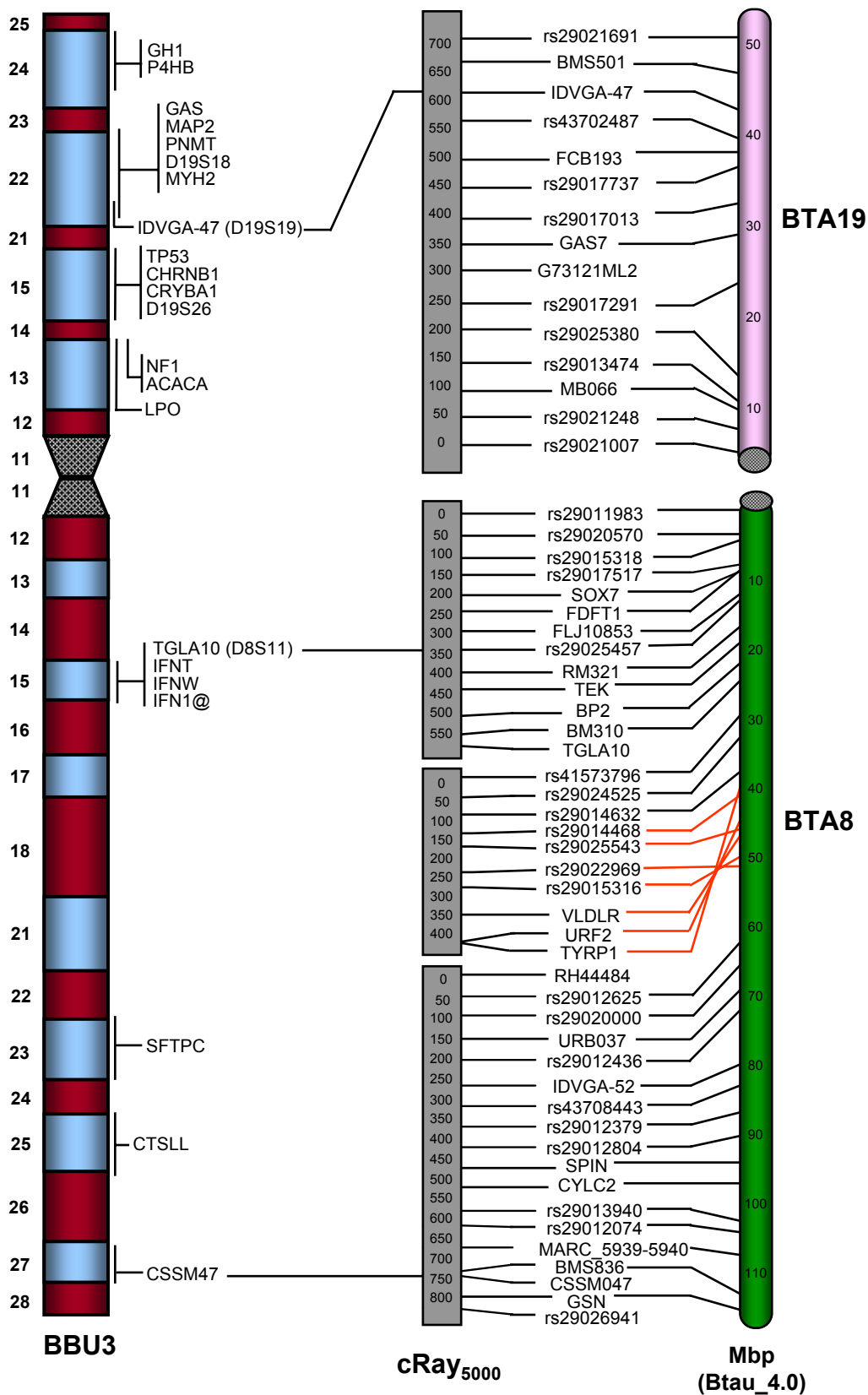

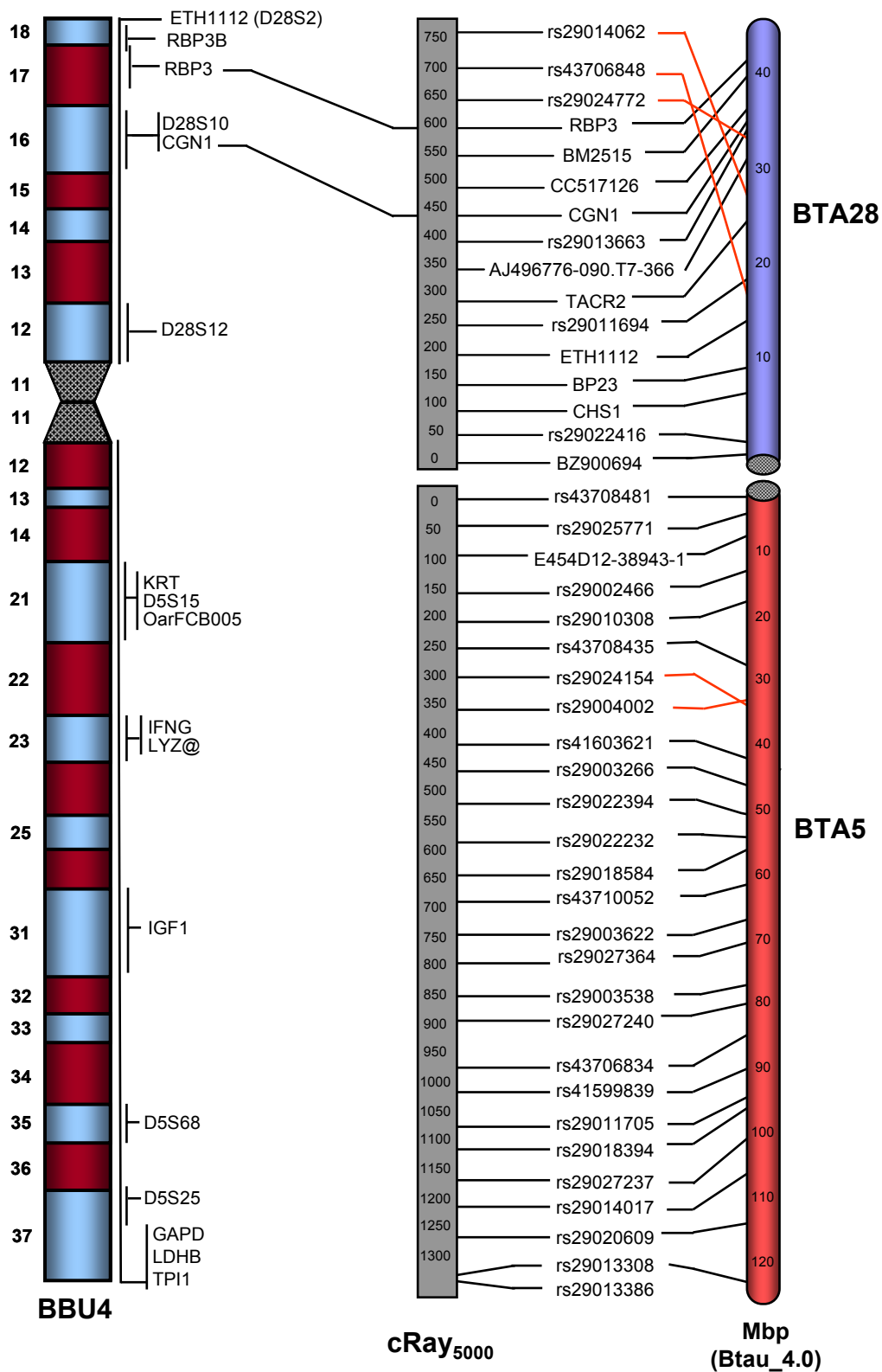

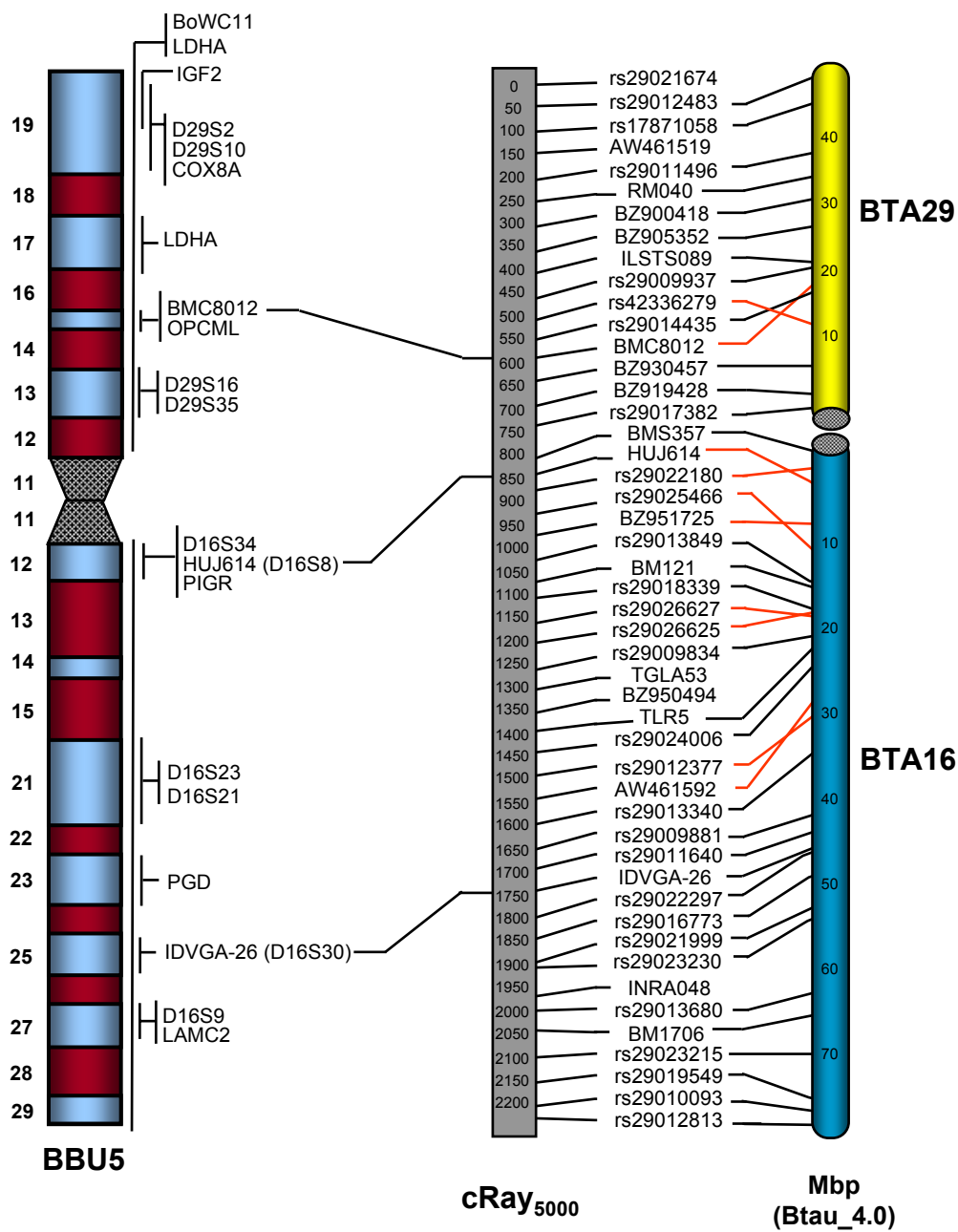

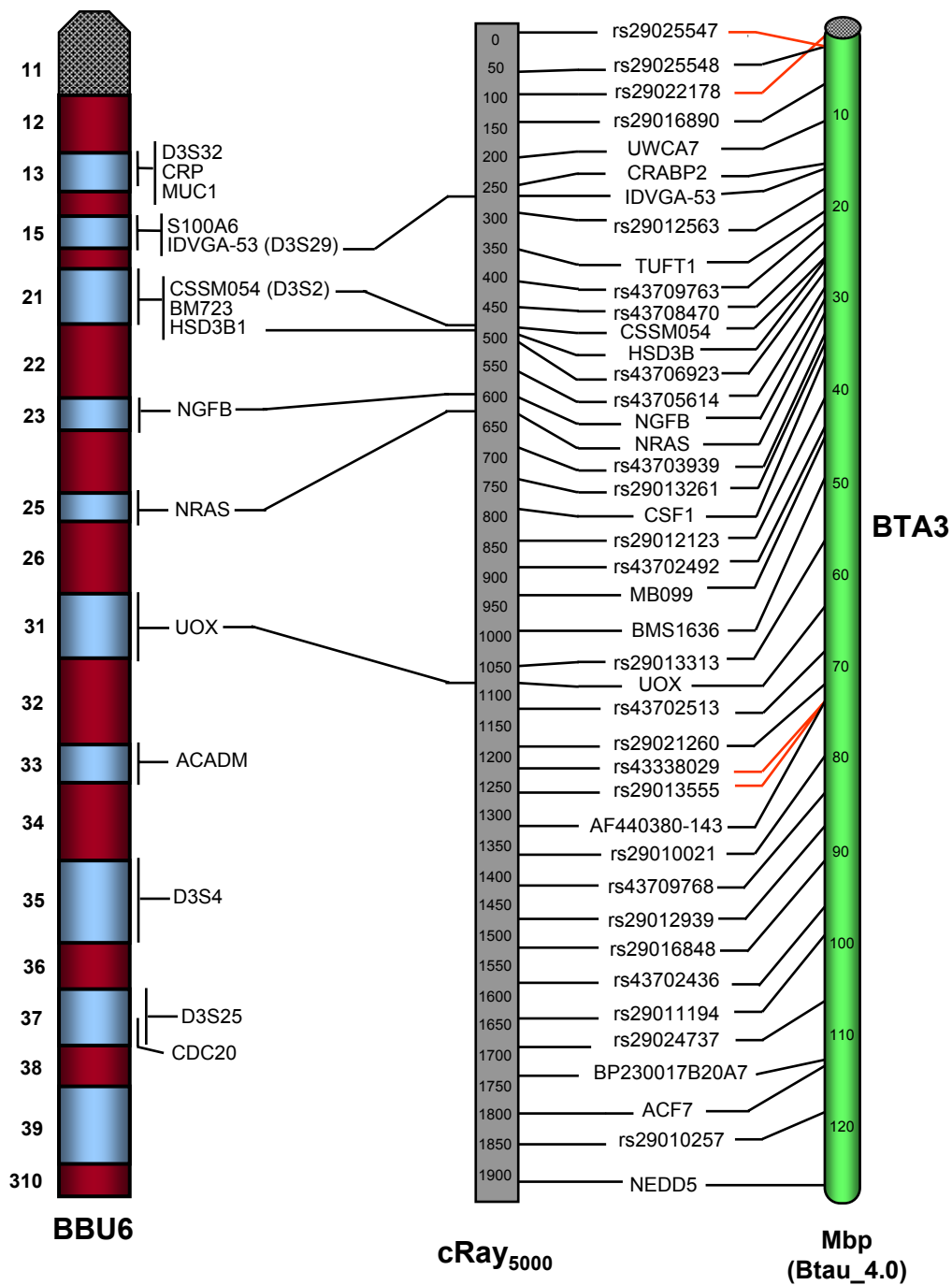

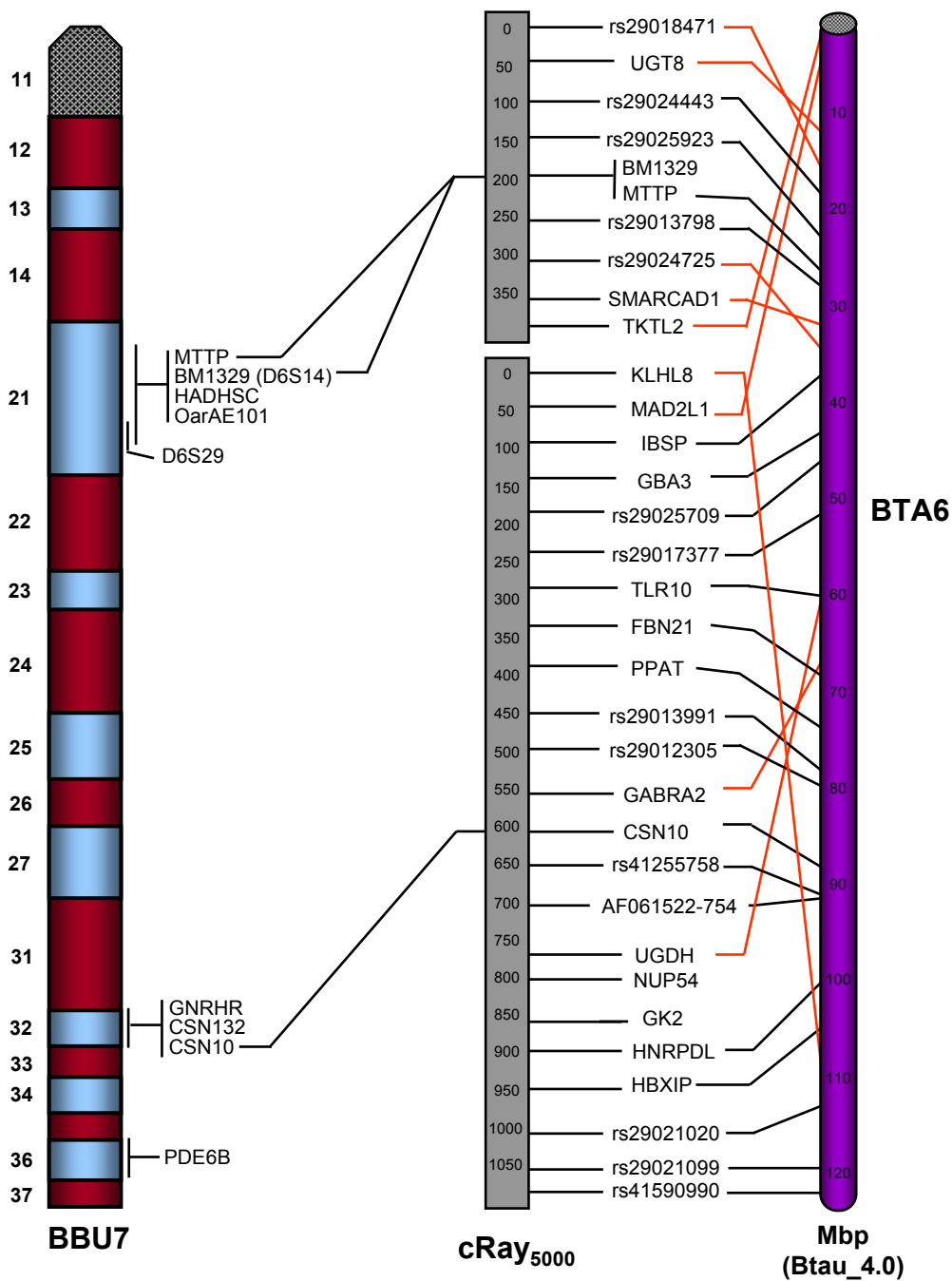

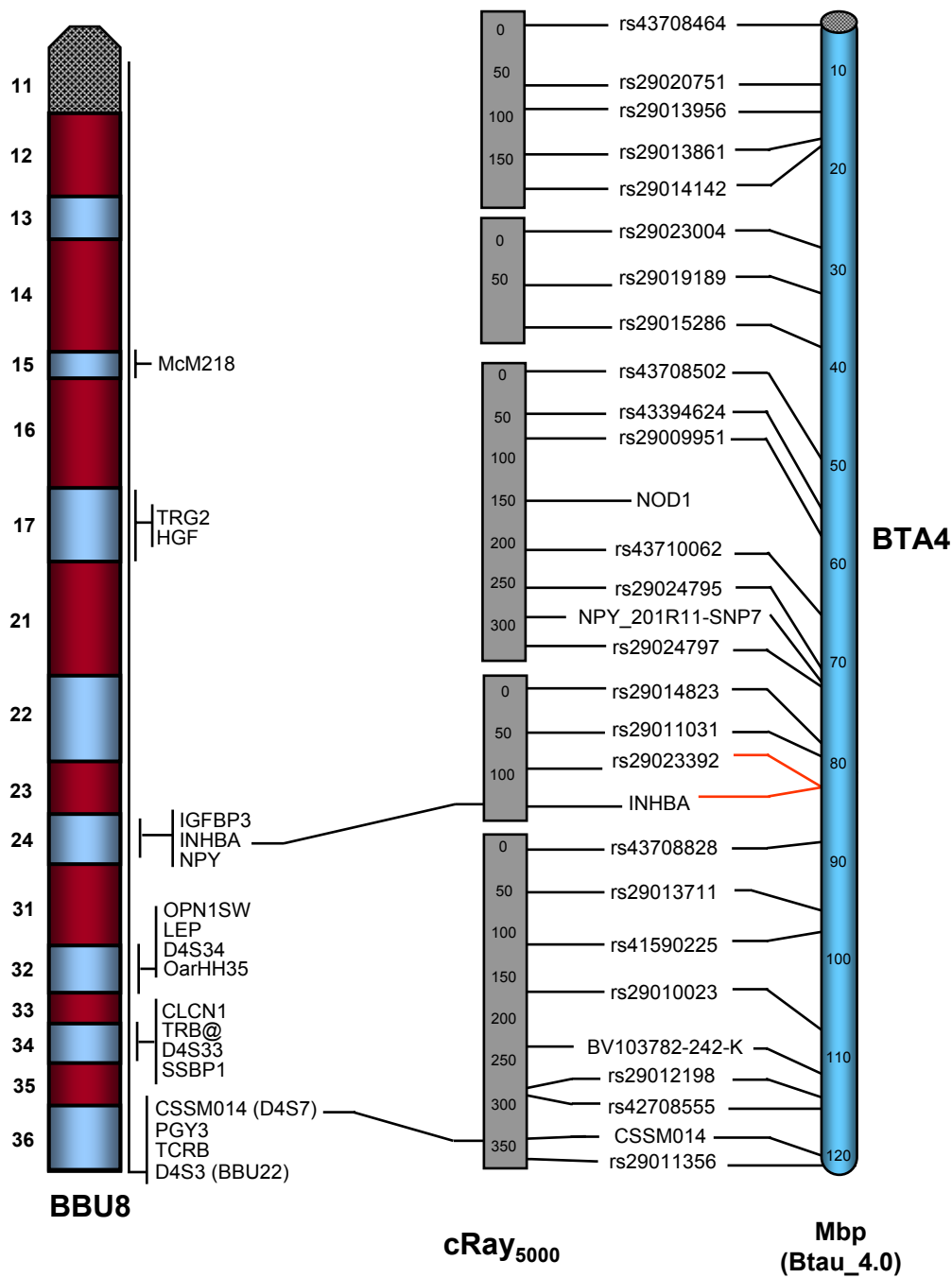

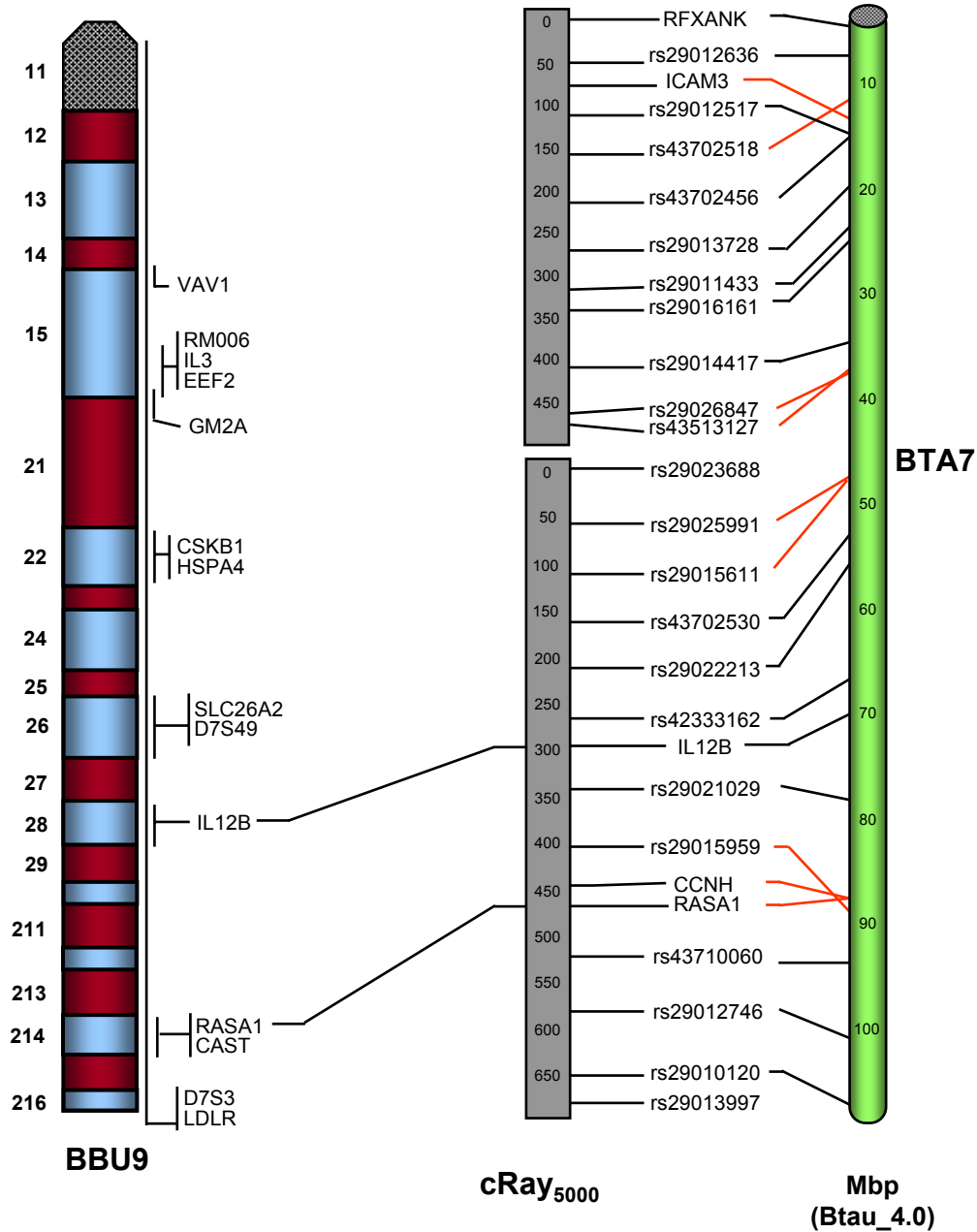

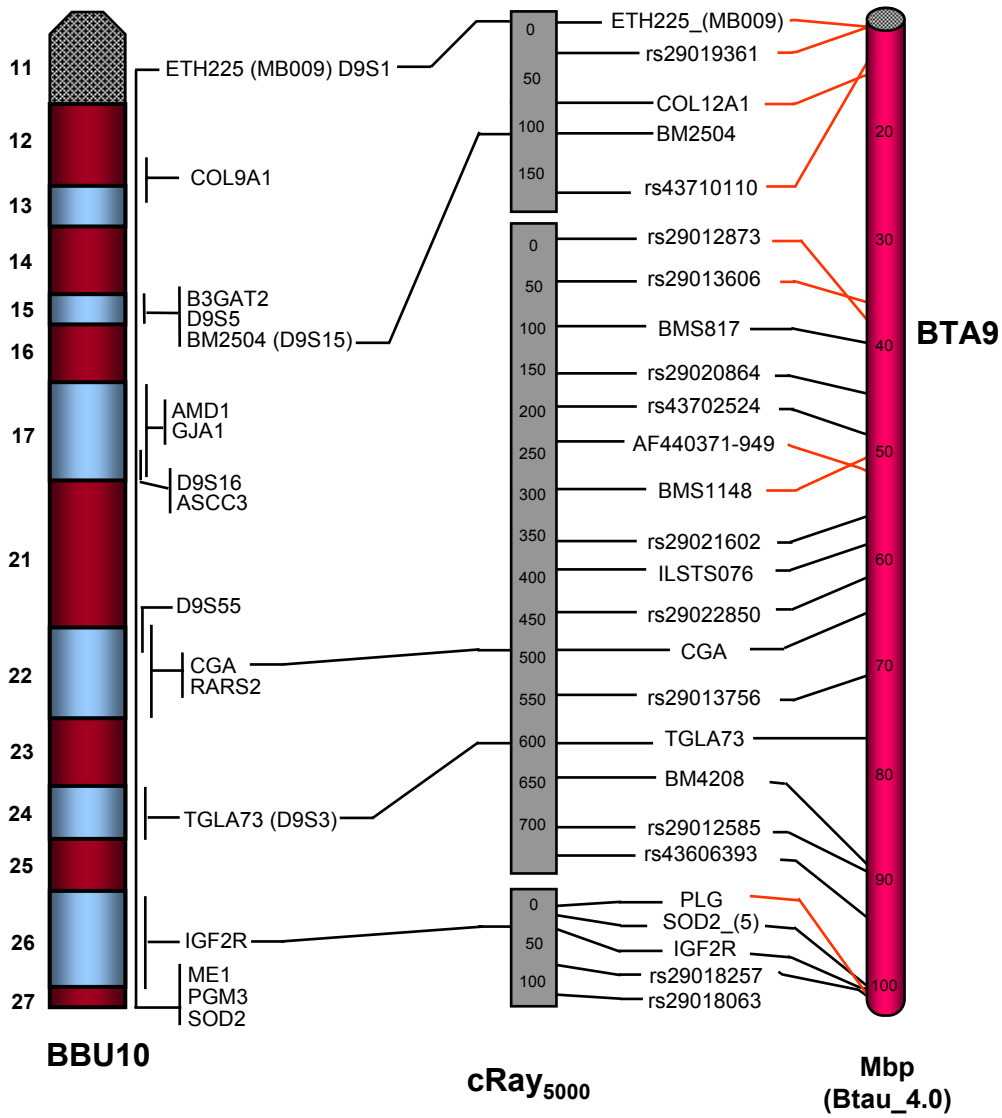

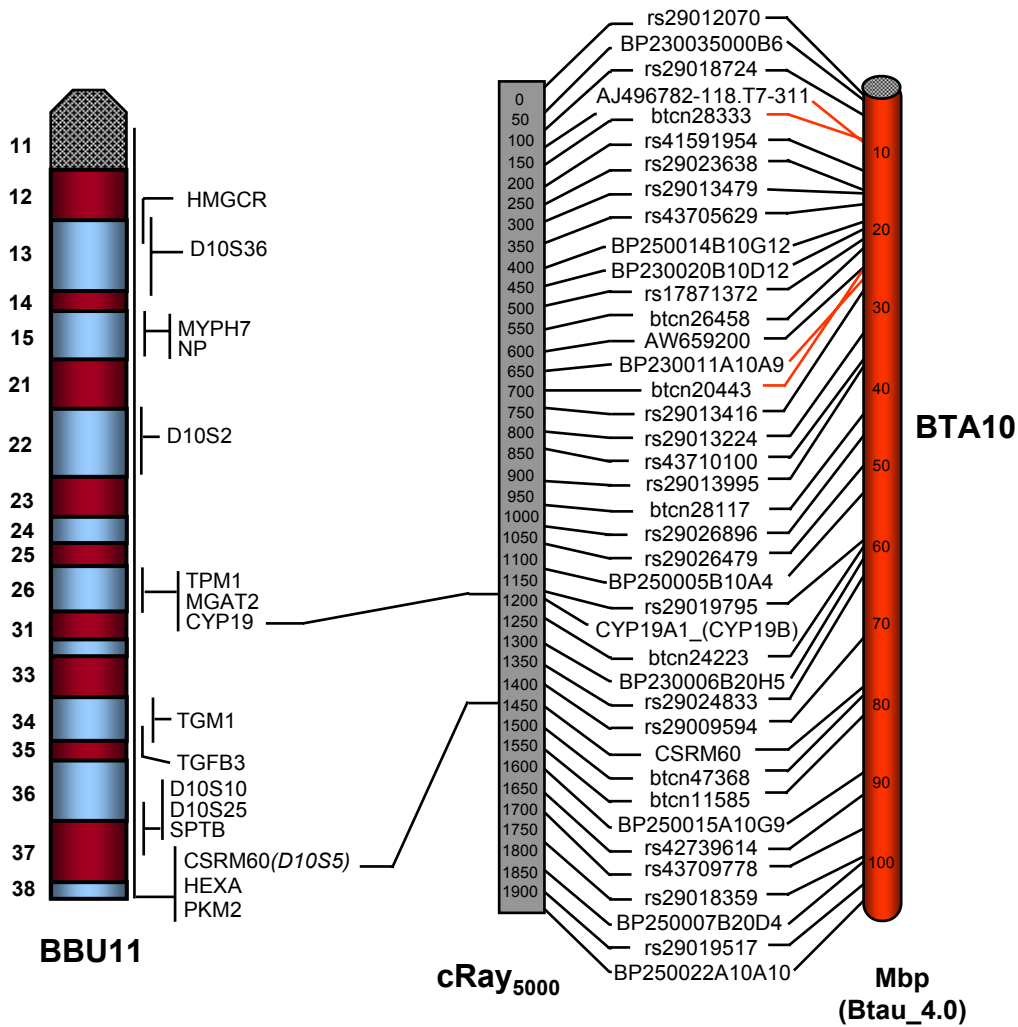

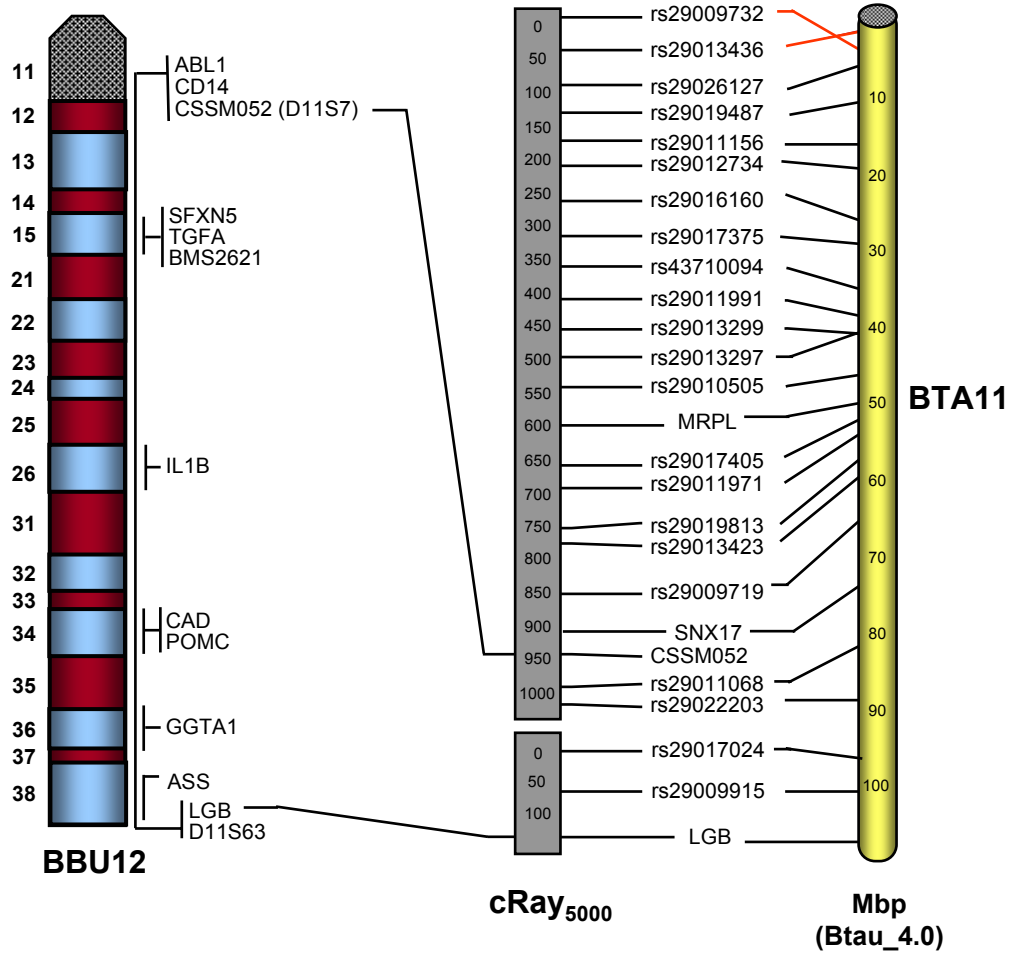

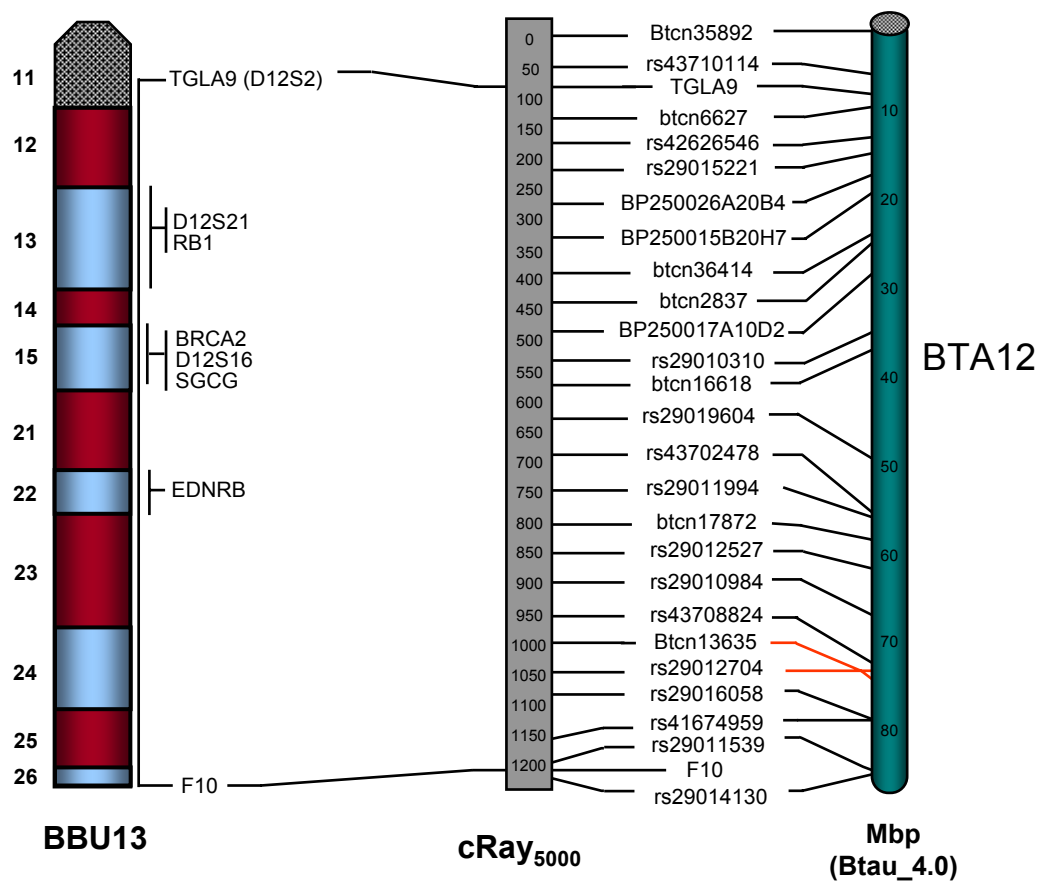

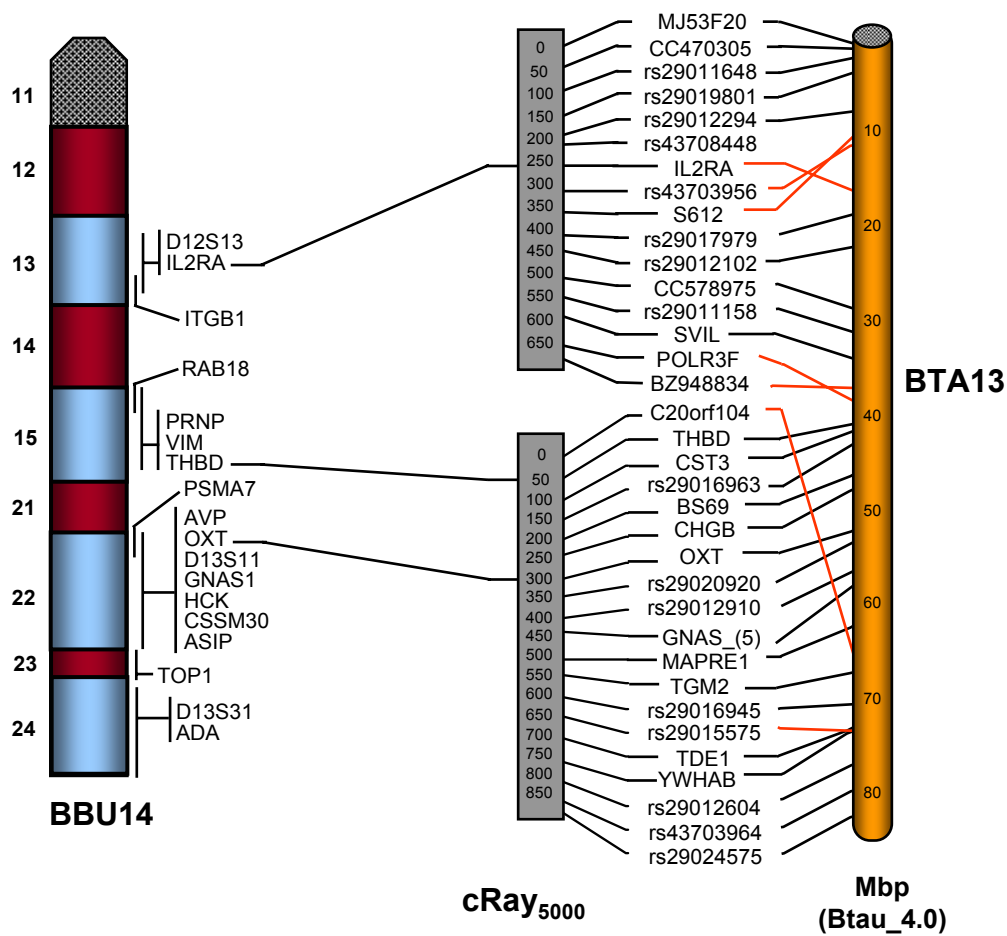

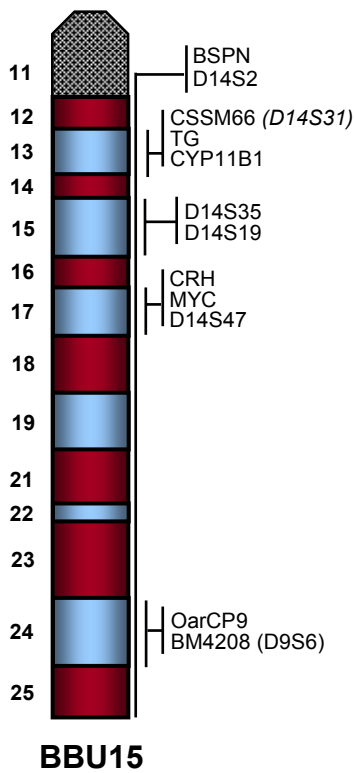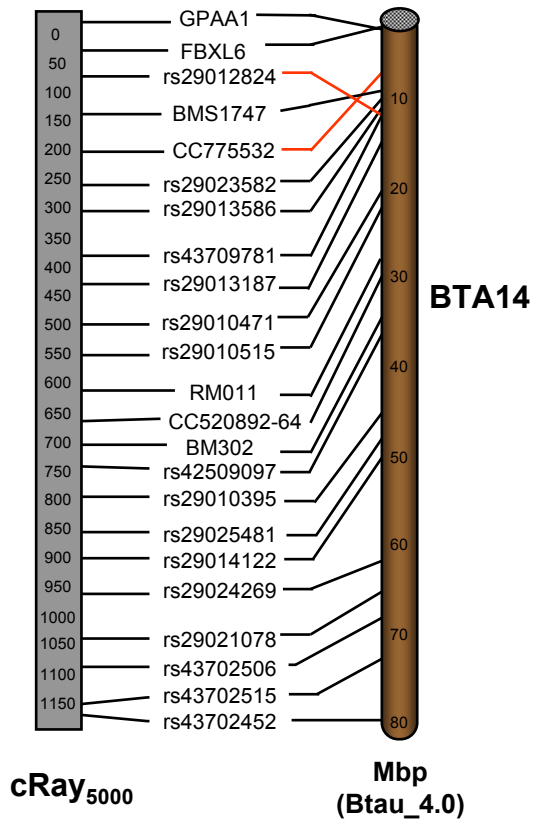

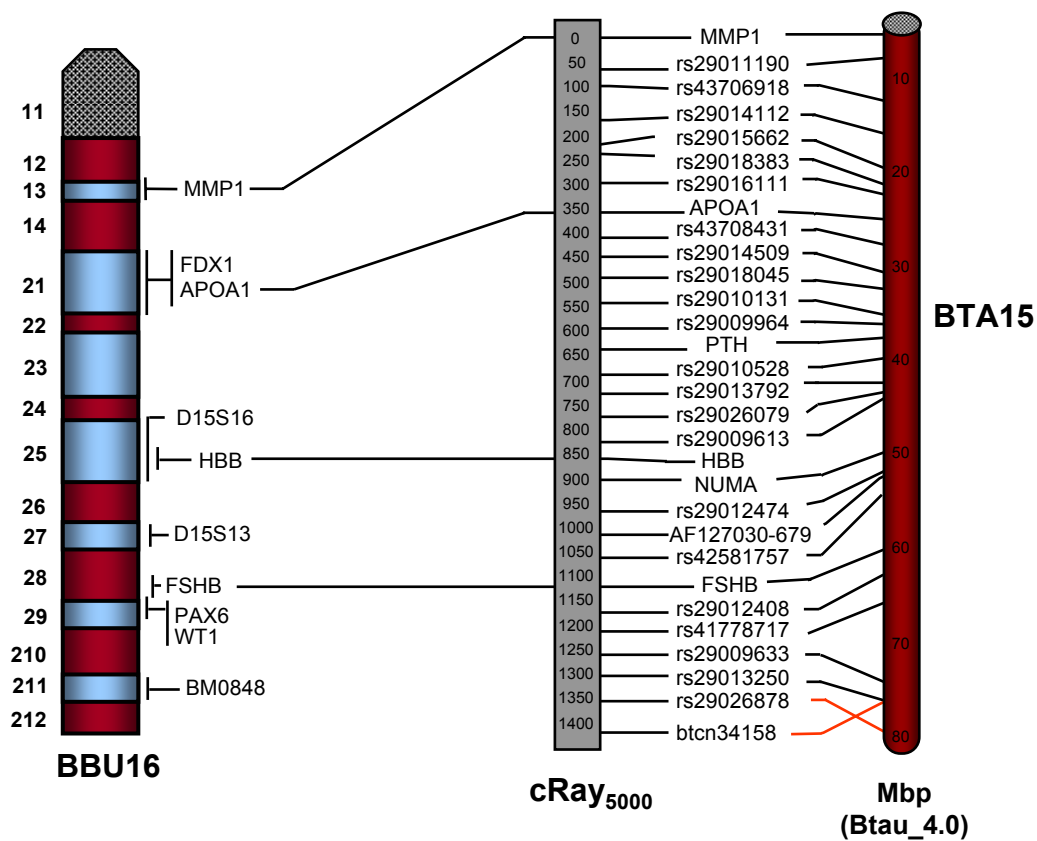

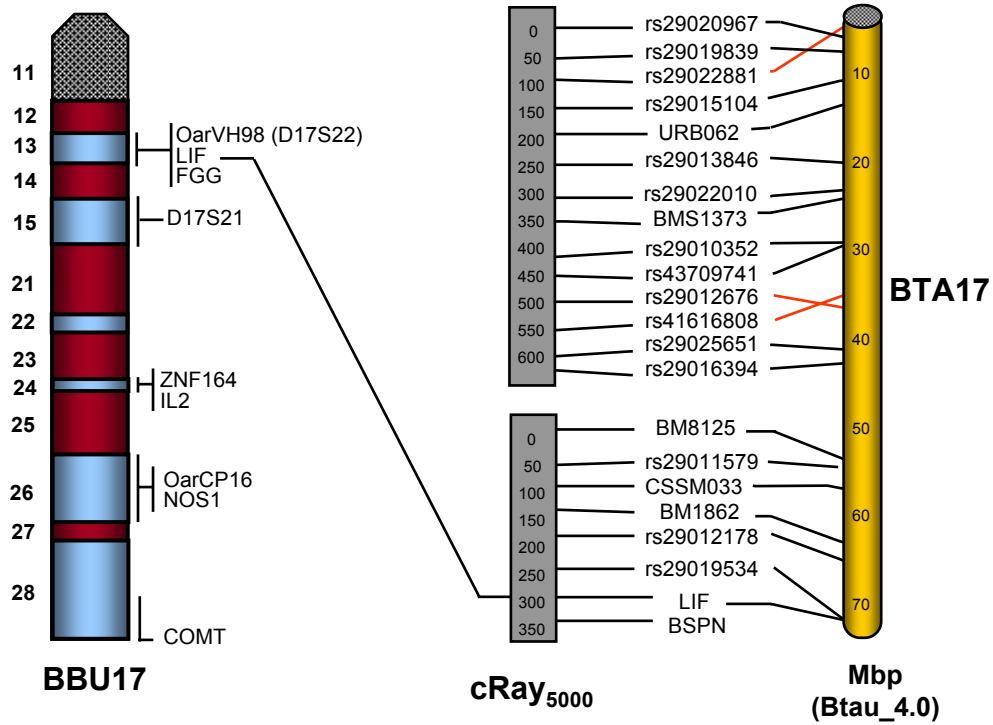

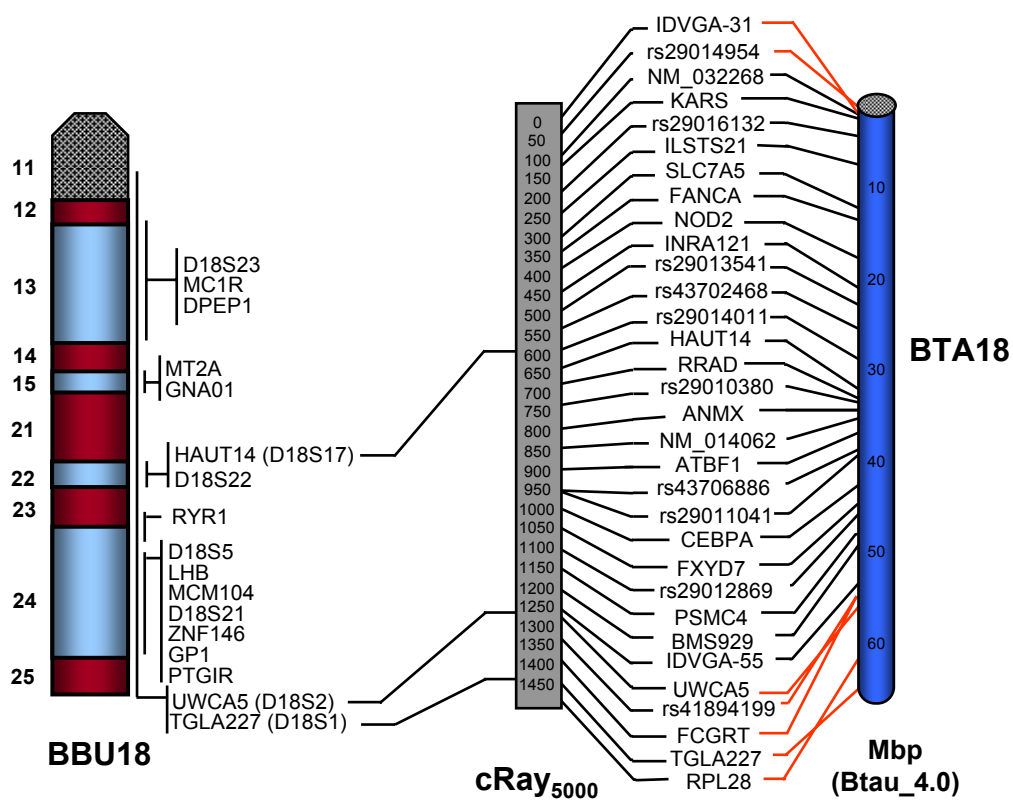

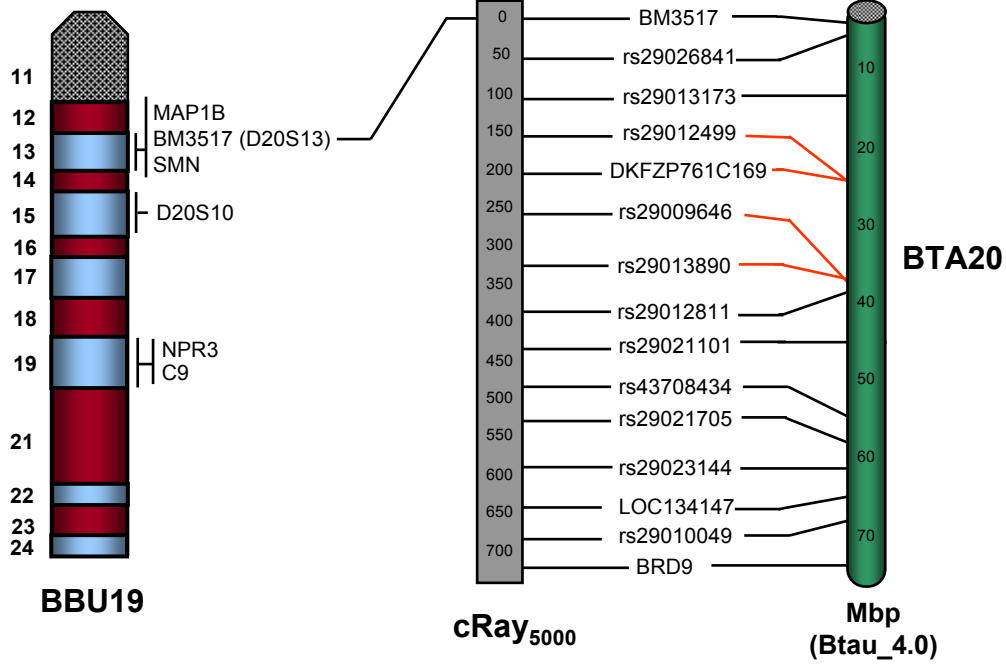

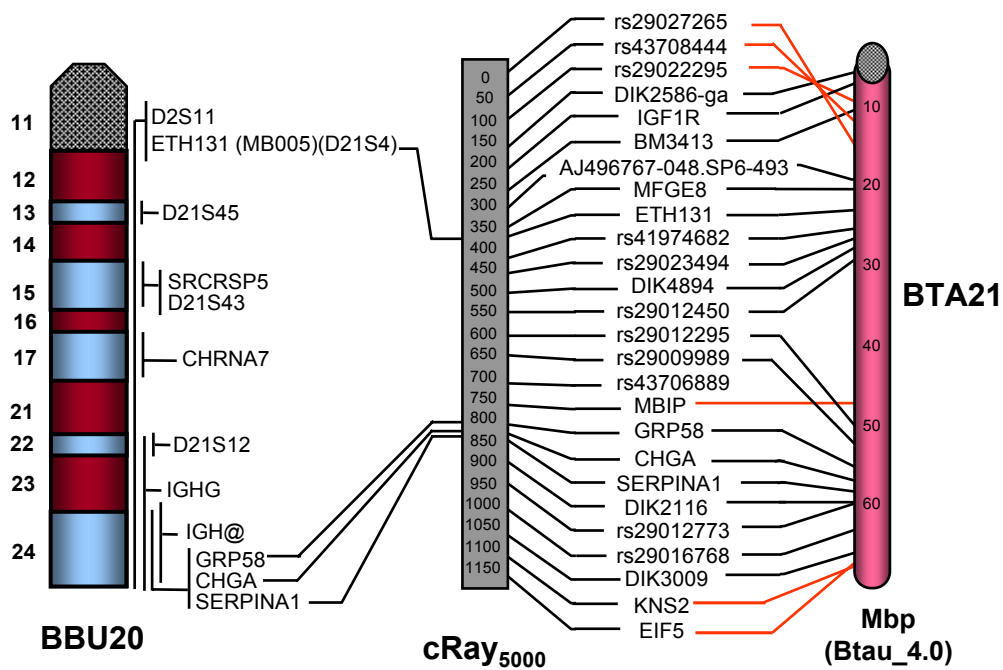

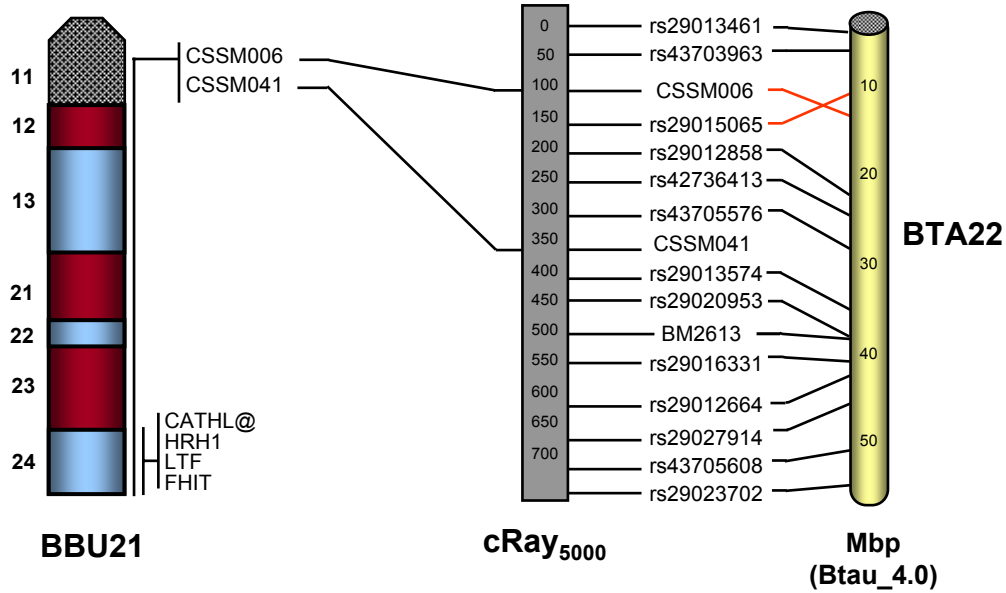

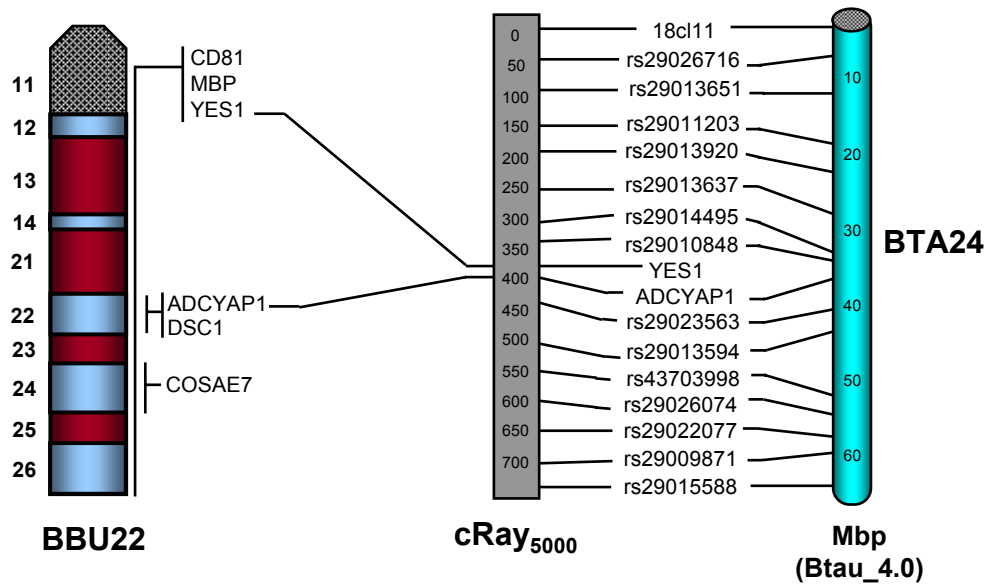

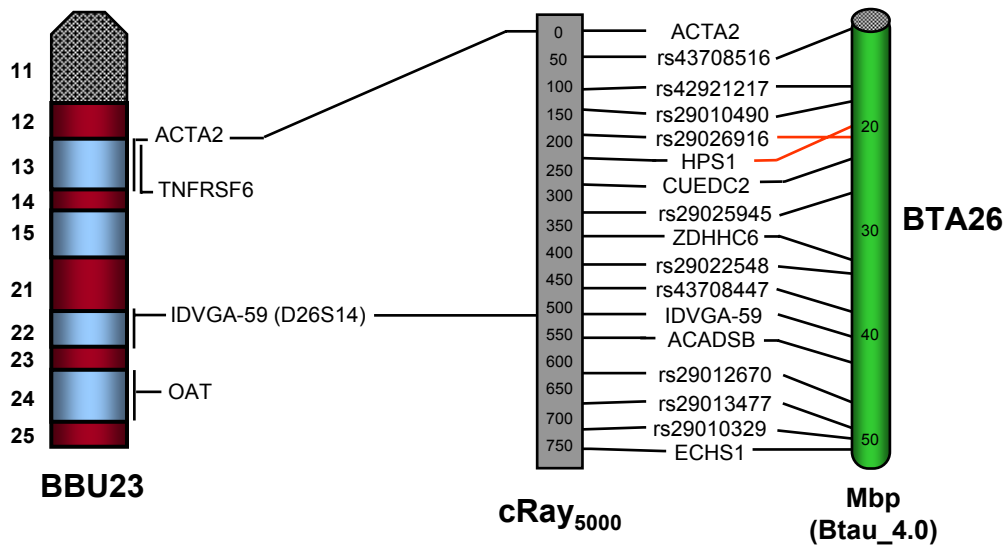

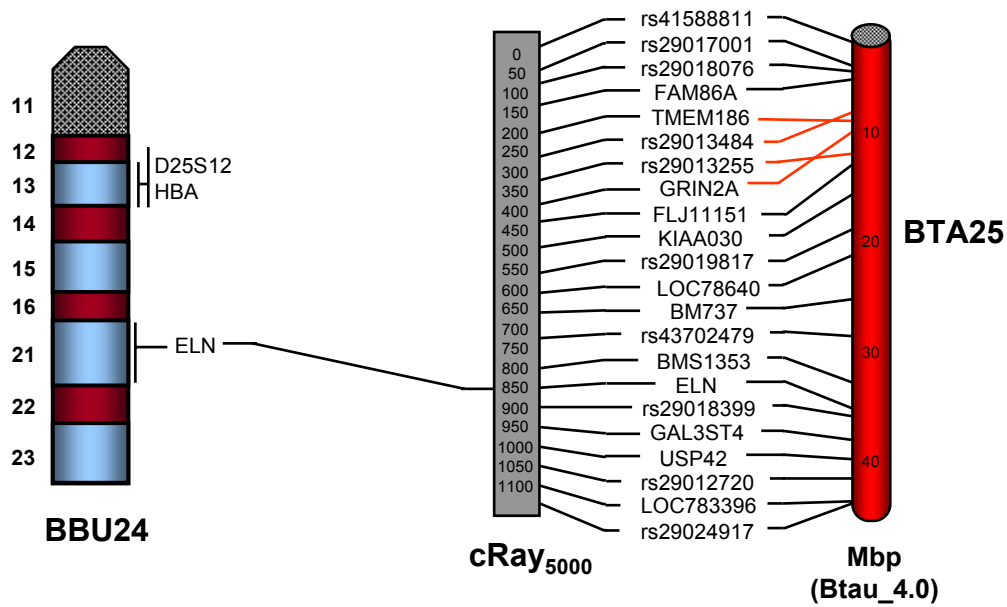

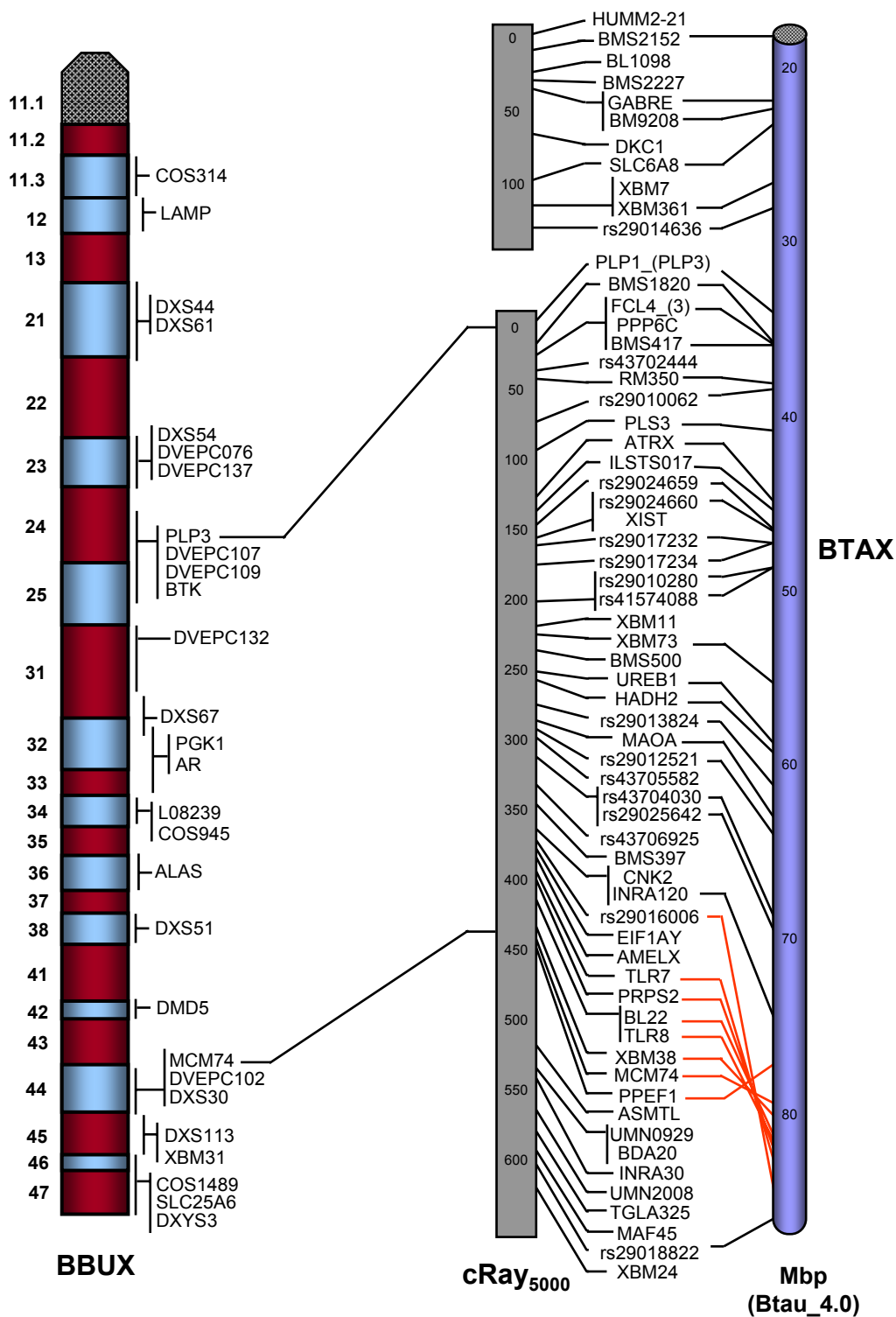

Supplement: Additional file 2 — Comparative maps from the buffalo genome (24 autosomes and the X chromosome), including the RH maps, the latest G-banded ideogram of the river buffalo chromosomes and the alignment with the bovine genome sequence assembly (Btau_4.0). The RH maps are shown in the centre, the G-banded ideogram on the left and the corresponding cattle chromosomes on the right. The distances in cR5000 and Mbp are shown below each corresponding map. For better illustration, the BBU RH maps of the autosomes shows one marker per 50 cR and the BTA sequence maps shows one marker every 10 Mbp. Markers common to both BBU RH and the cattle sequence are joined by a solid black line or a solid red line. Solid red lines indicate markers which are oriented sequentially regarding the cattle but inverted. A solid black line also joins those markers on the BBU RH map that have been physically mapped by FISH to their location on the ideogram (Di Meo et al. 2008). [file 1471-2164-9-631-S2.pdf]
