# Supplementary material for: A first generation whole genome RH map of the river buffalo with comparison to domestic cattle
Source: BMC Genomics. 2008 Dec 24;9:631. doi: 10.1186/1471-2164-9-631 (PMC2625372; doi:10.1186/1471-2164-9-631)
Supplement: Additional file 3 — Identification numbers of the markers displayed on NCBI/Probe database. In the file, first column is ProbeDB identification number, second column is submission's accession number, third column is submission's version and fourth column is markers tracking names. [file 1471-2164-9-631-S3.pdf]

9691634	2651	1	ADAM2	*
9691635	2651	1	ADAMTS1	*
9691636	2651	1	AHSG	*
9691637	2651	1	AW267109	*
9691638	2651	1	BBX	*
9691639	2651	1	BM1312	*
9691640	2651	1	BM1856	*
9691641	2651	1	BM6438	*
9691642	2651	1	BM6506	*
9691643	2651	1	BM6526	*
9691644	2651	1	BMS2263	*
9691645	2651	1	BMS4030	*
9691646	2651	1	BMS527	*
9691647	2651	1	BRF2	*
9691648	2651	1	C21orf45	*
9691649	2651	1	C8orf42	*
9691650	2651	1	C8orf79	*
9691651	2651	1	CARF	*
9691652	2651	1	CASR	*
9691653	2651	1	CLN8	*
9691654	2651	1	CNOT7	*
9691655	2651	1	CRYAA	*
9691656	2651	1	CSSM019	*
9691657	2651	1	DCTN6	*
9691658	2651	1	DEFB1	*
9691659	2651	1	DLGAP2	*
9691660	2651	1	DOC1	*
9691661	2651	1	FOXL2	*
9691662	2651	1	HLCS	*
9691663	2651	1	HOOK3	*
9691664	2651	1	IFNAR1	*
9691665	2651	1	IFNGR2	*
9691666	2651	1	IL12A	*
9691667	2651	1	LOC151584	*
9691668	2651	1	MX1	*
9691669	2651	1	NCK1	*
9691670	2651	1	NRG1	*
9691671	2651	1	ODZ3	*
9691672	2651	1	PDIR	*
9691673	2651	1	PLAT	*
9691674	2651	1	POU1F1	*
9691675	2651	1	PPP1R2	*
9691676	2651	1	PROS1	*
9691677	2651	1	PRSS7	*
9691678	2651	1	RM095	*
9691679	2651	1	RM209	*
9691680	2651	1	RNF7	*
9691681	2651	1	ROBO2	*
9691682	2651	1	SH2D4A	*
9691683	2651	1	SLC25A4	*
9691684	2651	1	SOD1	*
9691685	2651	1	SR140	*
9691686	2651	1	STCH5	*
9691687	2651	1	TACTILE	*
9691688	2651	1	TGLA179	*
9691689	2651	1	TGLA57	*
9691690	2651	1	THAP1	*
9691691	2651	1	TLOC1	*
9691692	2651	1	TRAD	*
9691693	2651	1	WRN	*
9691694	2651	1	ALCAM	*
9691695	2651	1	APOD	*
9691696	2651	1	ESDN	*
9691697	2651	1	INRA134	*
9691698	2651	1	KRTAP8	*
9691699	2651	1	TGLA49	*

9691700	2651	1	ACTR3	*
9691701	2651	1	ARHGEF4	*
9691702	2651	1	BM3627	*
9691703	2651	1	BMS4036	*
9691704	2651	1	BoLA-DMA	*
9691705	2651	1	BoLA-DNA	*
9691706	2651	1	BoLA-DQA2	*
9691707	2651	1	BoLA-DRA	*
9691708	2651	1	BoLA-DRB	*
9691709	2651	1	BoLA-DRB2	*
9691710	2651	1	BoLA-DRB3	*
9691711	2651	1	BY11	*
9691712	2651	1	BZ921457	*
9691713	2651	1	BZ953683	*
9691714	2651	1	C6ORF136	*
9691715	2651	1	CASP8	*
9691716	2651	1	CCND3	*
9691717	2651	1	CD28	*
9691718	2651	1	CLIC1	*
9691719	2651	1	COL3A1-95	*
9691720	2651	1	CXCR4	*
9691721	2651	1	DOM3Z	*
9691722	2651	1	FBXO9	*
9691723	2651	1	GCLC	*
9691724	2651	1	GDF8	*
9691725	2651	1	GORASP2	*
9691726	2651	1	GRB14	*
9691727	2651	1	GSTA2	*
9691728	2651	1	GUCA1A	*
9691729	2651	1	HLA-DMB	*
9691730	2651	1	HRB	*
9691731	2651	1	INHA	*
9691732	2651	1	ITPR3	*
9691733	2651	1	LOC510454	*
9691734	2651	1	MARC_21447-21448	*
9691735	2651	1	MARC_21564-21565	*
9691736	2651	1	MARC_4031-4032	*
9691737	2651	1	MARC_5369-5370	*
9691738	2651	1	MUT	*
9691739	2651	1	NXPH2	*
9691740	2651	1	PEX6	*
9691741	2651	1	POLR1C	*
9691742	2651	1	PRL	*
9691743	2651	1	PTP4A2	*
9691744	2651	1	RH18135	*
9691745	2651	1	SCG2	*
9691746	2651	1	SHGC-31988	*
9691747	2651	1	SKIV2L	*
9691748	2651	1	SLC11A1	*
9691749	2651	1	SMARCAL	*
9691750	2651	1	STAM2	*
9691751	2651	1	STAT1	*
9691752	2651	1	TAPBP	*
9691753	2651	1	TEXAN-2	*
9691754	2651	1	TGLA44	*
9691755	2651	1	TNRC5	*
9691756	2651	1	TTN-1	*
9691757	2651	1	WASF2	*
9691758	2651	1	WRNIP1	*
9691759	2651	1	GCG	*
9691760	2651	1	MTCH1	*
9691761	2651	1	TGLA377	*
9691762	2651	1	ACVR1	*
9691763	2651	1	ALPL	*
9691764	2651	1	BMON115	*
9691765	2651	1	BoLA-DQA	*

9691766	2651	1	BoLA-DYA	*
9691767	2651	1	BoLA-DYB	*
9691768	2651	1	CFLAR	*
9691769	2651	1	D2S1630E	*
9691770	2651	1	FLJ3277	*
9691771	2651	1	NUDC	*
9691772	2651	1	PSMB9	*
9691773	2651	1	ACADVL	*
9691774	2651	1	ATP6V1G1	*
9691775	2651	1	BLK	*
9691776	2651	1	CHIRUC2	*
9691777	2651	1	CNP	*
9691778	2651	1	CYLC2	*
9691779	2651	1	FDFT1	*
9691780	2651	1	G73127	*
9691781	2651	1	GSN	*
9691782	2651	1	LPL	*
9691783	2651	1	LZTS1	*
9691784	2651	1	MARC_5939-5940	*
9691785	2651	1	MARC_7851-7852	*
9691786	2651	1	MYH1	*
9691787	2651	1	P4HB	*
9691788	2651	1	TEK	*
9691789	2651	1	TYRP1	*
9691790	2651	1	VLDLR	*
9691791	2651	1	XKR6	*
9691792	2651	1	BE096286	*
9691793	2651	1	COL1A1	*
9691794	2651	1	CSAP16E	*
9691795	2651	1	PMC201071P3	*
9691796	2651	1	RH44484	*
9691797	2651	1	SRCRS10	*
9691798	2651	1	TPJ2	*
9691799	2651	1	ANK3	*
9691800	2651	1	ANXA11	*
9691801	2651	1	ANXA8	*
9691802	2651	1	AW464595	*
9691803	2651	1	BL25	*
9691804	2651	1	BM2515	*
9691805	2651	1	BMC1002	*
9691806	2651	1	BMC6020	*
9691807	2651	1	BMS1714	*
9691808	2651	1	BMS2079	*
9691809	2651	1	BMS2658	*
9691810	2651	1	BMS362	*
9691811	2651	1	BP23	*
9691812	2651	1	BZ900694	*
9691813	2651	1	CC472414	*
9691814	2651	1	CC504182	*
9691815	2651	1	CC517126	*
9691816	2651	1	CC537389	*
9691817	2651	1	CHS1	*
9691818	2651	1	ETH1112	*
9691819	2651	1	IDVGA-43	*
9691820	2651	1	ILSTS060	*
9691821	2651	1	PPYR1	*
9691822	2651	1	RBP3	*
9691823	2651	1	TACR2	*
9691824	2651	1	ZNF33A	*
9691825	2651	1	BMS697	*
9691826	2651	1	HK1	*
9691827	2651	1	AW267148	*
9691828	2651	1	AW289394	*
9691829	2651	1	AW461592	*
9691830	2651	1	AW482289	*
9691831	2651	1	BM121	*

```
9691832 2651   1 BM1706   *
9691833 2651   1 BMC3224  *
9691834 2651   1 BMC6004  *
9691835 2651   1 BMC8012  *
9691836 2651   1 BMS357   *
9691837 2651   1 BMS462   *
9691838 2651   1 BZ900418 *
9691839 2651   1 BZ901563 *
9691840 2651   1 BZ905352 *
9691841 2651   1 BZ910068 *
9691842 2651   1 BZ930457 *
9691843 2651   1 BZ932751 *
9691844 2651   1 BZ933262 *
9691845 2651   1 BZ938148 *
9691846 2651   1 BZ944687 *
9691847 2651   1 BZ946384 *
9691848 2651   1 BZ948508 *
9691849 2651   1 BZ950494 *
9691850 2651   1 BZ951039 *
9691851 2651   1 BZ951725 *
9691852 2651   1 BZ952478 *
9691853 2651   1 CC447793 *
9691854 2651   1 CSSM003  *
9691855 2651   1 HUJ614   *
9691856 2651   1 IDVGA-26 *
9691857 2651   1 ILSTS019 *
9691858 2651   1 ILSTS057 *
9691859 2651   1 ILSTS081 *
9691860 2651   1 ILSTS089 *
9691861 2651   1 RM040    *
9691862 2651   1 BZ919428 *
9691863 2651   1 BZ919809 *
9691864 2651   1 BZ945895 *
9691865 2651   1 AW289372 *
9691866 2651   1 AW461519 *
9691867 2651   1 BMS1600  *
9691868 2651   1 CC562100 *
9691869 2651   1 INRA048  *
9691870 2651   1 RME33    *
9691871 2651   1 S68957   *
9691872 2651   1 ACF7     *
9691873 2651   1 AW267062 *
9691874 2651   1 BE217553 *
9691875 2651   1 BL41     *
9691876 2651   1 BMS1636  *
9691877 2651   1 BMS2075  *
9691878 2651   1 BMS835   *
9691879 2651   1 BMS963   *
9691880 2651   1 CRABP2   *
9691881 2651   1 CSF1     *
9691882 2651   1 CSSM054  *
9691883 2651   1 CYP2J2   *
9691884 2651   1 HSD3B    *
9691885 2651   1 IDVGA-27 *
9691886 2651   1 IDVGA-53 *
9691887 2651   1 INRA041  *
9691888 2651   1 JUN      *
9691889 2651   1 MB099    *
9691890 2651   1 MUF1     *
9691891 2651   1 NCDN     *
9691892 2651   1 NEDD5    *
9691893 2651   1 NGFB     *
9691894 2651   1 NRAS     *
9691895 2651   1 OVGP1    *
9691896 2651   1 PGM1     *
9691897 2651   1 PTGFR    *
```

9691898	2651	1	SAG	*
9691899	2651	1	TUFT1	*
9691900	2651	1	UOX	*
9691901	2651	1	UWCA7	*
9691902	2651	1	VAV3	*
9691903	2651	1	DPYD	*
9691904	2651	1	EDG1	*
9691905	2651	1	MB101	*
9691906	2651	1	RM019	*
9691907	2651	1	ABCG2	*
9691908	2651	1	ACOX3	*
9691909	2651	1	AGXT2L1	*
9691910	2651	1	ATP8A1	*
9691911	2651	1	BM1236	*
9691912	2651	1	BM415	*
9691913	2651	1	BM4621	*
9691914	2651	1	BMS382	*
9691915	2651	1	CCKAR	*
9691916	2651	1	CENPC1	*
9691917	2651	1	CENTD1	*
9691918	2651	1	CHIC2	*
9691919	2651	1	CNOT6L	*
9691920	2651	1	CSN10	*
9691921	2651	1	CSN3	*
9691922	2651	1	DIK5359	*
9691923	2651	1	DIK5407	*
9691924	2651	1	DMP1	*
9691925	2651	1	EPHA5	*
9691926	2651	1	FBN12	*
9691927	2651	1	FBN14	*
9691928	2651	1	FBN21	*
9691929	2651	1	GABRA2	*
9691930	2651	1	GBA3	*
9691931	2651	1	GPR103	*
9691932	2651	1	HBXIP	*
9691933	2651	1	HNRPDL	*
9691934	2651	1	IBSP	*
9691935	2651	1	IGFBP7	*
9691936	2651	1	KDR	*
9691937	2651	1	KIAA0746	*
9691938	2651	1	KLHL8	*
9691939	2651	1	LAP3	*
9691940	2651	1	LDB2	*
9691941	2651	1	LPHN3	*
9691942	2651	1	LRPAP1	*
9691943	2651	1	MAD2L1	*
9691944	2651	1	MAPK10	*
9691945	2651	1	MTTP	*
9691946	2651	1	NCAPG	*
9691947	2651	1	NDST4	*
9691948	2651	1	NEUROG2	*
9691949	2651	1	PHOX2B	*
9691950	2651	1	PI4K2B	*
9691951	2651	1	PPAT	*
9691952	2651	1	PPP3CA	*
9691953	2651	1	PTPN13-LGC	*
9691954	2651	1	QDPR	*
9691955	2651	1	SCARB2	*
9691956	2651	1	SLIT2	*
9691957	2651	1	SMARCAD1	*
9691958	2651	1	SPP1	*
9691959	2651	1	TKTL2	*
9691960	2651	1	TLR10	*
9691961	2651	1	TLR6	*
9691962	2651	1	TRAM1L1	*
9691963	2651	1	UBE2D3	*

9691964	2651	1	UGDH	*	
9691965	2651	1	UGT8	*	
9691966	2651	1	UNC5C	*	
9691967	2651	1	GK2	*	
9691968	2651	1	BM1329	*	
9691969	2651	1	BM143	*	
9691970	2651	1	BMC4203	*	
9691971	2651	1	CXCL1	*	
9691972	2651	1	FBN13	*	
9691973	2651	1	NUP54	*	
9691974	2651	1	OARHH55	*	
9691975	2651	1	PPARGC1A	*	
9691976	2651	1	SNCA	*	
9691977	2651	1	SOD3	*	
9691978	2651	1	TEC	*	
9691979	2651	1	TLR1	*	
9691980	2651	1	CSSM014	*	
9691981	2651	1	INHBA	*	
9691982	2651	1	CCNH	*	
9691983	2651	1	COMP	*	
9691984	2651	1	CRTL1	*	
9691985	2651	1	D7S3	*	
9691986	2651	1	GUK1		*
9691987	2651	1	ICAM3		*
9691988	2651	1	IL12B		*
9691989	2651	1	IL4		*
9691990	2651	1	MEF2C		*
9691991	2651	1	RASA1		*
9691992	2651	1	RFXANK		*
9691993	2651	1	IRF1		*
9691994	2651	1	BM4208		*
9691995	2651	1	BMS1148		*
9691996	2651	1	BMS1234		*
9691997	2651	1	BMS1290		*
9691998	2651	1	BMS1724		*
9691999	2651	1	BMS1909		*
9692000	2651	1	BMS817		*
9692001	2651	1	CGA		*
9692002	2651	1	COL12A1		*
9692003	2651	1	ETH225_(MB009)		*
9692004	2651	1	IGF2R		*
9692005	2651	1	ILSTS076		*
9692006	2651	1	MLLT4		*
9692007	2651	1	MYB		*
9692008	2651	1	PLG		*
9692009	2651	1	PLN		*
9692010	2651	1	SOD2_(5)		*
9692011	2651	1	TGLA73		*
9692012	2651	1	VIL2		*
9692013	2651	1	PPP6C		*
9692014	2651	1	BMS2377		*
9692015	2651	1	BMS555		*
9692016	2651	1	CTGF		*
9692017	2651	1	REN49736		*
9692018	2651	1	ADAM29		*
9692019	2651	1	AW466107		*
9692020	2651	1	AW659200		*
9692021	2651	1	BE589327		*
9692022	2651	1	BF774993		*
9692023	2651	1	BP230005B10F10		*
9692024	2651	1	BP230006B20H5		*
9692025	2651	1	BP230010A10C2		*
9692026	2651	1	BP230010B20B9		*
9692027	2651	1	BP230011A10A9		*
9692028	2651	1	BP230012B20F8		*
9692029	2651	1	BP230020B10D12		*

9692030	2651	1	BP230021A10E3	*
9692031	2651	1	BP230035000B6	*
9692032	2651	1	BP230036000E3	*
9692033	2651	1	BP240034A10G4	*
9692034	2651	1	BP250002A20F1	*
9692035	2651	1	BP250002B20G8	*
9692036	2651	1	BP250005B10A4	*
9692037	2651	1	BP250007B20D4	*
9692038	2651	1	BP250008A10D12	*
9692039	2651	1	BP250012A20G8	*
9692040	2651	1	BP250014B10G12	*
9692041	2651	1	BP250015A10A10	*
9692042	2651	1	BP250015A10G9	*
9692043	2651	1	BP250015A10H10	*
9692044	2651	1	BP250015B10E9	*
9692045	2651	1	BP250015B20B2	*
9692046	2651	1	BP250022A10A10	*
9692047	2651	1	BP250022B20B1	*
9692048	2651	1	btcn11585	*
9692049	2651	1	btcn20443	*
9692050	2651	1	btcn21374	*
9692051	2651	1	btcn22649	*
9692052	2651	1	Btcn24223	*
9692053	2651	1	btcn24758	*
9692054	2651	1	btcn26458	*
9692055	2651	1	btcn28117	*
9692056	2651	1	btcn28333	*
9692057	2651	1	btcn34158	*
9692058	2651	1	btcn36177	*
9692059	2651	1	btcn39699	*
9692060	2651	1	btcn41123	*
9692061	2651	1	btcn4374	*
9692062	2651	1	btcn45788	*
9692063	2651	1	btcn47368	*
9692064	2651	1	CSRM60	*
9692065	2651	1	Btcn35777	*
9692066	2651	1	CYP19A1_(CYP19B)	*
9692067	2651	1	BTBD6	*
9692068	2651	1	EIF5	*
9692069	2651	1	IGHA1	*
9692070	2651	1	KNS2	*
9692071	2651	1	LGB	*
9692072	2651	1	SNX17	*
9692073	2651	1	UTP14A	*
9692074	2651	1	CSSM052	*
9692075	2651	1	BP230017B20A7	*
9692076	2651	1	BP230035000C2	*
9692077	2651	1	BP250001B10D6	*
9692078	2651	1	BP250002A10D5	*
9692079	2651	1	BP250007A20A12	*
9692080	2651	1	BP250008A10E9	*
9692081	2651	1	BP250010A20H9	*
9692082	2651	1	BP250015A10A2	*
9692083	2651	1	BP250015B20H7	*
9692084	2651	1	BP250017A10D2	*
9692085	2651	1	BP250023B10E7	*
9692086	2651	1	BP250026A20B4	*
9692087	2651	1	btcn12770	*
9692088	2651	1	Btcn13635	*
9692089	2651	1	btcn16618	*
9692090	2651	1	btcn16640	*
9692091	2651	1	btcn1695	*
9692092	2651	1	btcn17872	*
9692093	2651	1	btcn18594	*
9692094	2651	1	btcn20796	*
9692095	2651	1	btcn2277	*

```
9692096 2651   1 btcn2837  *
9692097 2651   1 btcn33592 *
9692098 2651   1 Btcn34811 *
9692099 2651   1 Btcn35892 *
9692100 2651   1 btcn36414 *
9692101 2651   1 btcn38688 *
9692102 2651   1 btcn39170 *
9692103 2651   1 btcn40273 *
9692104 2651   1 btcn41665 *
9692105 2651   1 btcn6627  *
9692106 2651   1 TGLA9     *
9692107 2651   1 btcn44908 *
9692108 2651   1 F10       *
9692109 2651   1 ADA       *
9692110 2651   1 B4GALT5   *
9692111 2651   1 BPI       *
9692112 2651   1 BS69      *
9692113 2651   1 BZ908049  *
9692114 2651   1 BZ948834  *
9692115 2651   1 C10orf7   *
9692116 2651   1 C20orf104 *
9692117 2651   1 CC470305  *
9692118 2651   1 CC578975 *
9692119 2651   1 CHGB     *
9692120 2651   1 CRFG     *
9692121 2651   1 CST3     *
9692122 2651   1 DNAJC5   *
9692123 2651   1 GNAS_(5) *
9692124 2651   1 IDH3B    *
9692125 2651   1 IL2RA    *
9692126 2651   1 MAPRE1   *
9692127 2651   1 MAVS     *
9692128 2651   1 MJ53F20  *
9692129 2651   1 NCOA5    *
9692130 2651   1 NET1     *
9692131 2651   1 OXT      *
9692132 2651   1 PLCB4    *
9692133 2651   1 POLR3F   *
9692134 2651   1 PROCR    *
9692135 2651   1 PTGIS    *
9692136 2651   1 S612     *
9692137 2651   1 SNRPB2   *
9692138 2651   1 SVIL     *
9692139 2651   1 TDE1     *
9692140 2651   1 TGM2     *
9692141 2651   1 THBD     *
9692142 2651   1 YWHAB    *
9692143 2651   1 COMMD3   *
9692144 2651   1 PLCG1    *
9692145 2651   1 PRNP     *
9692146 2651   1 BM2934   *
9692147 2651   1 BM302    *
9692148 2651   1 BM4305   *
9692149 2651   1 BM4630   *
9692150 2651   1 BM6425   *
9692151 2651   1 BM8215   *
9692152 2651   1 BMC1207  *
9692153 2651   1 BMS108   *
9692154 2651   1 BMS1747  *
9692155 2651   1 BMS2055  *
9692156 2651   1 CSSM066  *
9692157 2651   1 DGAT1    *
9692158 2651   1 DIK_2439 *
9692159 2651   1 DIK_4015 *
9692160 2651   1 DIK_4177 *
9692161 2651   1 DIK_4182 *
```

```
9692162 2651   1 DIK_4681 *
9692163 2651   1 DIK_4707 *
9692164 2651   1 DIK_4774 *
9692165 2651   1 DIK062   *
9692166 2651   1 FBXL6    *
9692167 2651   1 GPAA1    *
9692168 2651   1 HSF      *
9692169 2651   1 ILSTS008 *
9692170 2651   1 ILSTS039 *
9692171 2651   1 INRA092  *
9692172 2651   1 MAF      *
9692173 2651   1 PAG      *
9692174 2651   1 RM011    *
9692175 2651   1 RM137    *
9692176 2651   1 BM4513   *
9692177 2651   1 DIK_2592 *
9692178 2651   1 BM1508   *
9692179 2651   1 DIK_1101 *
9692180 2651   1 DIK_2196 *
9692181 2651   1 DIK_2201 *
9692182 2651   1 DIK_2447 *
9692183 2651   1 DIK_2742 *
9692184 2651   1 ILSTS011         *
9692185 2651   1 APOA1            *
9692186 2651   1 CD44             *
9692187 2651   1 FSHB             *
9692188 2651   1 MGC33371         *
9692189 2651   1 MMP1             *
9692190 2651   1 NUMA             *
9692191 2651   1 PORIMIN          *
9692192 2651   1 PTH              *
9692193 2651   1 RRM1             *
9692194 2651   1 HBB              *
9692195 2651   1 SDHD             *
9692196 2651   1 AL184253_(MMP11) *
9692197 2651   1 BM1862           *
9692198 2651   1 BM8125           *
9692199 2651   1 BMS1373          *
9692200 2651   1 CSSM033          *
9692201 2651   1 ETH185_(MB008)   *
9692202 2651   1 HUJ223           *
9692203 2651   1 LIF              *
9692204 2651   1 TGLA231          *
9692205 2651   1 UCP1             *
9692206 2651   1 URB062    *
9692207 2651   1 BZ917545  *
9692208 2651   1 CC500703  *
9692209 2651   1 IOZARA975 *
9692210 2651   1 OarVH98   *
9692211 2651   1 TLR2      *
9692212 2651   1 18cl1     *
9692213 2651   1 18cl11    *
9692214 2651   1 18cl2     *
9692215 2651   1 ADCK4     *
9692216 2651   1 AMFR      *
9692217 2651   1 ANMX      *
9692218 2651   1 ATBF1     *
9692219 2651   1 AURKC     *
9692220 2651   1 AXL       *
9692221 2651   1 BCKDHA    *
9692222 2651   1 BM2078    *
9692223 2651   1 BM6507    *
9692224 2651   1 BM7109    *
9692225 2651   1 BMS2785   *
9692226 2651   1 BMS929    *
9692227 2651   1 CA11      *
```

```
9692228 2651   1 CALB2    *
9692229 2651   1 CBFB     *
9692230 2651   1 CDH5     *
9692231 2651   1 CEBPA    *
9692232 2651   1 COX6B    *
9692233 2651   1 CYLD     *
9692234 2651   1 DMAHP    *
9692235 2651   1 DNCLI2   *
9692236 2651   1 ERCC2    *
9692237 2651   1 ETFB     *
9692238 2651   1 FANCA    *
9692239 2651   1 FCGRT    *
9692240 2651   1 FXYD7    *
9692241 2651   1 GALNS    *
9692242 2651   1 GMFG     *
9692243 2651   1 HAUT14   *
9692244 2651   1 HP       *
9692245 2651   1 HSD11B2  *
9692246 2651   1 IDVGA-31 *
9692247 2651   1 IDVGA-55 *
9692248 2651   1 ILSTS21  *
9692249 2651   1 INRA121  *
9692250 2651   1 IRF3      *
9692251 2651   1 KARS      *
9692252 2651   1 KIAA0174  *
9692253 2651   1 KLP1      *
9692254 2651   1 LCAT      *
9692255 2651   1 MAP4K1    *
9692256 2651   1 MMP2      *
9692257 2651   1 NM_014062 *
9692258 2651   1 NM_017803 *
9692259 2651   1 NM_024860 *
9692260 2651   1 NM_032268 *
9692261 2651   1 NOD2      *
9692262 2651   1 NQO1      *
9692263 2651   1 NUTF2     *
9692264 2651   1 PEG3      *
9692265 2651   1 PLD3      *
9692266 2651   1 POP4      *
9692267 2651   1 PSMB10    *
9692268 2651   1 PSMC4     *
9692269 2651   1 RPL28     *
9692270 2651   1 RRAD      *
9692271 2651   1 SF3B3     *
9692272 2651   1 SIRT2        *
9692273 2651   1 SLC7A5       *
9692274 2651   1 SPG7         *
9692275 2651   1 TGLA227      *
9692276 2651   1 TK2          *
9692277 2651   1 TNNT1        *
9692278 2651   1 UBA2         *
9692279 2651   1 UWCA5        *
9692280 2651   1 VPS4A        *
9692281 2651   1 NM_024519    *
9692282 2651   1 PPP2R1A      *
9692283 2651   1 SYMPK        *
9692284 2651   1 BM3517       *
9692285 2651   1 BRD9         *
9692286 2651   1 CCT5         *
9692287 2651   1 DKFZP761C169 *
9692288 2651   1 LOC134147    *
9692289 2651   1 MYO10        *
9692290 2651   1 ZNF262       *
9692291 2651   1 PHGDH        *
9692292 2651   1 BM3413       *
9692293 2651   1 BMC5221      *
```

```
9692294 2651   1 BMS2382    *
9692295 2651   1 CC350547   *
9692296 2651   1 CHGA       *
9692297 2651   1 DIK2116    *
9692298 2651   1 DIK2367    *
9692299 2651   1 DIK2821    *
9692300 2651   1 DIK2849    *
9692301 2651   1 DIK3001    *
9692302 2651   1 DIK3009    *
9692303 2651   1 DIK4322    *
9692304 2651   1 DIK4894    *
9692305 2651   1 ETH131     *
9692306 2651   1 GRP58      *
9692307 2651   1 IGF1R      *
9692308 2651   1 ILSTS054   *
9692309 2651   1 ILSTS092   *
9692310 2651   1 MBIP       *
9692311 2651   1 MFGE8      *
9692312 2651   1 MULGE4     *
9692313 2651   1 SERPINA1   *
9692314 2651   1 DIK2586-ga *
9692315 2651   1 AGLA233    *
9692316 2651   1 AKT1     *
9692317 2651   1 BMS1494  *
9692318 2651   1 BMS1561  *
9692319 2651   1 BMS868   *
9692320 2651   1 DIK3023  *
9692321 2651   1 IDVGA-39 *
9692322 2651   1 RM151    *
9692323 2651   1 BM1558   *
9692324 2651   1 BM2613   *
9692325 2651   1 BMS2573  *
9692326 2651   1 BMS672   *
9692327 2651   1 CSSM006  *
9692328 2651   1 CSSM026  *
9692329 2651   1 INRA194  *
9692330 2651   1 TLR9     *
9692331 2651   1 CSSM041  *
9692332 2651   1 HUJI75   *
9692333 2651   1 ADCYAP1  *
9692334 2651   1 YES1     *
9692335 2651   1 ACADSB   *
9692336 2651   1 CSL4     *
9692337 2651   1 CUEDC2   *
9692338 2651   1 ECHS1    *
9692339 2651   1 HIF1AN   *
9692340 2651   1 HPS1     *
9692341 2651   1 IDVGA-59 *
9692342 2651   1 SEC23IP  *
9692343 2651   1 XPNPEP1  *
9692344 2651   1 ZDHHC6   *
9692345 2651   1 ACTA2    *
9692346 2651   1 MGEA5    *
9692347 2651   1 APOB48R  *
9692348 2651   1 AQP8     *
9692349 2651   1 ARPC1A   *
9692350 2651   1 BM1864   *
9692351 2651   1 BM4005   *
9692352 2651   1 BM7207   *
9692353 2651   1 BM737    *
9692354 2651   1 BMC4216  *
9692355 2651   1 BMS1353  *
9692356 2651   1 BMS65    *
9692357 2651   1 C16Orf72 *
9692358 2651   1 CENTA1   *
9692359 2651   1 CHST12   *
```

```
9692360 2651   1 DAGLB      *
9692361 2651   1 ELN        *
9692362 2651   1 EMP2       *
9692363 2651   1 FAM86A     *
9692364 2651   1 FLJ11151   *
9692365 2651   1 FOXK1      *
9692366 2651   1 GAL3ST4    *
9692367 2651   1 GRIN2A     *
9692368 2651   1 GTF3C1     *
9692369 2651   1 KIAA030    *
9692370 2651   1 LOC511442  *
9692371 2651   1 LOC616782  *
9692372 2651   1 LOC783396  *
9692373 2651   1 LOC78640   *
9692374 2651   1 MRPS17     *
9692375 2651   1 MYH11      *
9692376 2651   1 NUBP2      *
9692377 2651   1 PHKG2      *
9692378 2651   1 RAB26      *
9692379 2651   1 RBBP6      *
9692380 2651   1 SEC14L5    *
9692381 2651   1 TMEM186    *
9692382 2651   1 UMOD          *
9692383 2651   1 USP42         *
9692384 2651   1 RUND2CA       *
9692385 2651   1 AF4           *
9692386 2651   1 AF5           *
9692387 2651   1 BMS2843       *
9692388 2651   1 RABL5         *
9692389 2651   1 URB036        *
9692390 2651   1 TLR3          *
9692391 2651   1 D1S4_(MAF46)  *
9692392 2651   1 ILSTS030      *
9692393 2651   1 BE217497      *
9692394 2651   1 BM6000        *
9692395 2651   1 BMC5012       *
9692396 2651   1 BMS1920       *
9692397 2651   1 BMS2503       *
9692398 2651   1 BMS501        *
9692399 2651   1 CLTC          *
9692400 2651   1 ETH12         *
9692401 2651   1 FCB193        *
9692402 2651   1 GAS7          *
9692403 2651   1 IDVGA-47      *
9692404 2651   1 ILSTS014 *
9692405 2651   1 LOC90799 *
9692406 2651   1 MB066    *
9692407 2651   1 MGC20398 *
9692408 2651   1 PECAM1   *
9692409 2651   1 PSMC5    *
9692410 2651   1 ARRB2    *
9692411 2651   1 BM9202   *
9692412 2651   1 BMS1069  *
9692413 2651   1 NSD1     *
9692414 2651   1 TAU      *
9692415 2651   1 TBCD     *
9692416 2651   1 AKNA     *
9692417 2651   1 BM310    *
9692418 2651   1 BM8129   *
9692419 2651   1 BMS1591  *
9692420 2651   1 BMS2629  *
9692421 2651   1 BMS836   *
9692422 2651   1 BP2      *
9692423 2651   1 CLU      *
9692424 2651   1 FLJ10853 *
9692425 2651   1 FLJ31810 *
```

```
9692426 2651   1 IDVGA-52 *
9692427 2651   1 MB065    *
9692428 2651   1 MSRA     *
9692429 2651   1 NDUFB6   *
9692430 2651   1 ORM1     *
9692431 2651   1 PNOC     *
9692432 2651   1 PTP9Q22  *
9692433 2651   1 RM321    *
9692434 2651   1 SPIN     *
9692435 2651   1 SRC259   *
9692436 2651   1 SRC276   *
9692437 2651   1 TLR4     *
9692438 2651   1 URB037   *
9692439 2651   1 URF2     *
9692440 2651   1 ZNF189   *
9692441 2651   1 BMS2196  *
9692442 2651   1 BM3419   *
9692443 2651   1 BMS2847  *
9692444 2651   1 BMS678   *
9692445 2651   1 CSSM037  *
9692446 2651   1 CSSM047  *
9692447 2651   1 NUDT2    *
9692448 2651   1 RIG-I    *
9692449 2651   1 RM372    *
9692450 2651   1 ROR2     *
9692451 2651   1 SEMA4D   *
9692452 2651   1 CGN1     *
9692453 2651   1 BM6121   *
9692454 2651   1 TGLA53   *
9692455 2651   1 TLR5     *
9692456 2651   1 DUSP4    *
9692457 2651   1 KIF13B   *
9692458 2651   1 MRPL     *
9692459 2651   1 SOX7     *
9692460 2651   1 ATRX     *
9692461 2651   1 BL22     *
9692462 2651   1 BM6017   *
9692463 2651   1 BM9208   *
9692464 2651   1 BMS1820  *
9692465 2651   1 BMS2152  *
9692466 2651   1 BMS2227  *
9692467 2651   1 BMS397   *
9692468 2651   1 BMS417   *
9692469 2651   1 BMS903   *
9692470 2651   1 BMS938       *
9692471 2651   1 CNK2         *
9692472 2651   1 DKC1         *
9692473 2651   1 ELF4         *
9692474 2651   1 FCL4_(3)     *
9692475 2651   1 GABRE        *
9692476 2651   1 HADH2        *
9692477 2651   1 HUMM2-21     *
9692478 2651   1 ILSTS017     *
9692479 2651   1 INRA120      *
9692480 2651   1 MAOA         *
9692481 2651   1 MCM74        *
9692482 2651   1 PLP1_(PLP3)  *
9692483 2651   1 PLS3         *
9692484 2651   1 PPEF1        *
9692485 2651   1 PRPS2        *
9692486 2651   1 RM350        *
9692487 2651   1 SLC6A8       *
9692488 2651   1 TGLA325      *
9692489 2651   1 THOC2_(5)    *
9692490 2651   1 TLR7         *
9692491 2651   1 TLR8         *
```

```
9692492 2651   1 TNFSF5       *
9692493 2651   1 UREB1        *
9692494 2651   1 XBM361       *
9692495 2651   1 XBM38        *
9692496 2651   1 XBM73        *
9692497 2651   1 AMELX        *
9692498 2651   1 ASMTL        *
9692499 2651   1 BDA20        *
9692500 2651   1 BL1098       *
9692501 2651   1 BMS500       *
9692502 2651   1 INRA30       *
9692503 2651   1 MAF45        *
9692504 2651   1 XBM11        *
9692505 2651   1 XBM24        *
9692506 2651   1 XBM411       *
9692507 2651   1 XBM7         *
9692508 2651   1 XIST         *
9692509 2651   1 PBBRR1-0907r *
9692510 2651   1 TSPY         *
9692511 2651   1 UMN0304      *
9692512 2651   1 UMN0929      *
9692513 2651   1 UMN2008      *
9692514 2651   1 Yp11_Rep *
9692515 2651   1 AMELY    *
9692516 2651   1 BRY.1    *
9692517 2651   1 EIF1AY   *
9692518 2651   1 INRA189  *
9692519 2651   1 UMN0803  *
9692520 2651   1 UMN2908  *
```
